# Lysine Triggers Acute Oviposition by Activating the 20E-ETH-JH Signaling Cascade in *Drosophila melanogaster*

**DOI:** 10.3390/ijms27115065

**Published:** 2026-06-03

**Authors:** Siran Yu, Gang Zhou, Xiaolu Wang, Liming Zhang, Ping Li

**Affiliations:** 1 School of Life Sciences and Technology, Tongji University, Shanghai 200092, China; 2400023@sumhs.edu.cn (S.Y.); 2110823@tongji.edu.cn (G.Z.); 2311493@tongji.edu.cn (X.W.); 2333130@tongji.edu.cn (L.Z.); 2 College of Public Health, Shanghai University of Medicine and Health Sciences, Shanghai 201318, China

**Keywords:** egg laying, holidic medium, lysine, 20E–ETH–JH

## Abstract

Reproductive performance is closely linked to nutrient availability, yet the specific nutrient-derived cues governing oviposition decision-making remain inadequately defined. Chemically defined holidic media used in *Drosophila melanogaster* studies provide precise control over dietary components; however, the mechanisms by which individual nutrients exert acute effects on inter-organ endocrine signaling to trigger oviposition behavior are not well understood. In a two-choice oviposition assay, where substrates are presented within the same chamber, we observed that HM induces a rapid increase in egg laying in *D. melanogaster* compared to grape/apple juice media (GJM), yielding 4.02× and 5.27× more eggs at 2 h and 8 h, respectively. Systematic nutrient omission and subsequent supplementation identify lysine as the key essential amino acid driving this rapid oviposition response. Notably, this phenomenon reflects a short-term oviposition reaction, rather than long-term nutritional modulation of ovarian development. Lysine supplementation results in elevated levels of 20-hydroxyecdysone (20E) and juvenile hormone (JH), as well as the induction of ecdysis-triggering hormone (ETH) expression. Pharmacological interventions support a model in which a 20E–ETH–JH endocrine cascade underlies the lysine-dependent phenotype: methoprene phenocopies the effect, whereas precocene suppresses the HM-induced increase in egg laying. A 20E analog similarly enhances oviposition. Consistent with the role of JH in reproductive tissues, activation of the JH pathway correlates with increased expression of extracellular matrix genes in the ovarian muscle. Collectively, these results demonstrate that lysine acts as a critical nutritional cue to activate the 20E–ETH–JH endocrine axis and acutely promote egg laying behavior in *D. melanogaster* over a short time period. This provides a defined framework for investigating the short-term nutrient hormone regulation of ovulation and offers a practical strategy for efficient egg collection.

## 1. Introduction

Reproductive output in insects is influenced by both the physiological state of the female and the local environment at the time of egg deposition. In *Drosophila melanogaster*, oviposition site selection is an active decision-making process influenced by multiple sensory modalities [1,2,3]. Females evaluate substrate mechanics, olfactory cues, and contact-dependent gustatory cues when choosing where to lay eggs. For example, substrate stiffness is assessed through mechanosensory pathways, whereas odor cues such as citrus volatiles, microbial geosmin, and parasitoid-derived semiochemicals can promote or suppress oviposition depending on environmental context. Amino-acid-related cues may also influence feeding and egg-laying behavior through contact-dependent chemosensory pathways, including ionotropic receptor-mediated taste circuits. Therefore, oviposition preference for a nutrient-containing medium may reflect a combination of nutritional value, odor, taste, and physical substrate properties [4,5,6,7,8,9,10].

Most oviposition assays and egg-collection workflows in *D. melanogaster* utilize agar plates supplemented with grape or apple juice (hereafter GJM) [8,11]. While convenient, the undefined composition of GJM obscures nutrient-specific effects and complicates mechanistic analysis. Chemically defined holidic media (HM) address this limitation by enabling the systematic removal or reintroduction of individual nutrients [12]. Using HM, long-term deprivation of essential amino acids (EAAs) nearly abolishes egg laying, whereas depletion of other nutrient classes results in slower declines [8,12,13].

Most nutritional manipulations are assessed over several days, primarily focusing on changes in oogenesis and ovarian growth. However, whether specific nutrients can acutely modulate egg laying within hours—and through which inter-organ signals—remains unclear. Additionally, nutrients influence oviposition decision: females exhibit a preference for softer substrates, but nutritional cues can override mechanical avoidance [3]. Females spend more time and lay more eggs on amino acid-enriched substrates [14]; however, it remains unresolved whether amino acids exert their effects through rapid endocrine regulation of the reproductive tract, through changes in residence time driven by feeding, or through both mechanisms.

Endocrine circuits provide a plausible route for short-timescale nutrient control of egg laying. Juvenile hormone (JH) is a key gonadotropic hormone in adult insects and promotes ovulation and egg production; in *D. melanogaster*, JH signaling in the ovarian muscle induces extracellular matrix (ECM) genes such as laminins that are required for efficient ovulation [15]. Ecdysis-triggering hormone (ETH), released from Inka cells, acts as an allatotropin that stimulates JH synthesis in the corpus allatum (CA) [16,17,18,19,20,21,22]. Upstream, the steroid hormone 20-hydroxyecdysone (20E) can promote ETH synthesis and release, coupling steroid signaling to the ETH–JH axis [19,21,23]. Together, these findings suggest that nutrients might regulate egg-laying output by tuning a 20E–ETH–JH endocrine cascade.

This study utilizes HM to investigate the acute nutrient regulation of egg laying in *D. melanogaster*. A two-choice oviposition assay is established, enabling direct comparison of substrates within the same chamber. It is observed that HM supports significantly higher egg output within hours compared to GJM. Through systematic nutrient omission and single-amino acid supplementation, lysine is identified as the predominant EAA driving this acute response. Subsequently, transcriptomics, hormone measurements, and pharmacological perturbations are employed to support a model in which lysine activates a 20E–ETH–JH axis, accompanied by increased expression of ECM-related genes in the ovarian muscle, collectively facilitating rapid egg deposition.

## 2. Results

### 2.1. A Chemically Defined Holidic Medium Drives Rapid Egg Laying in D. melanogaster

**Figure 1:**
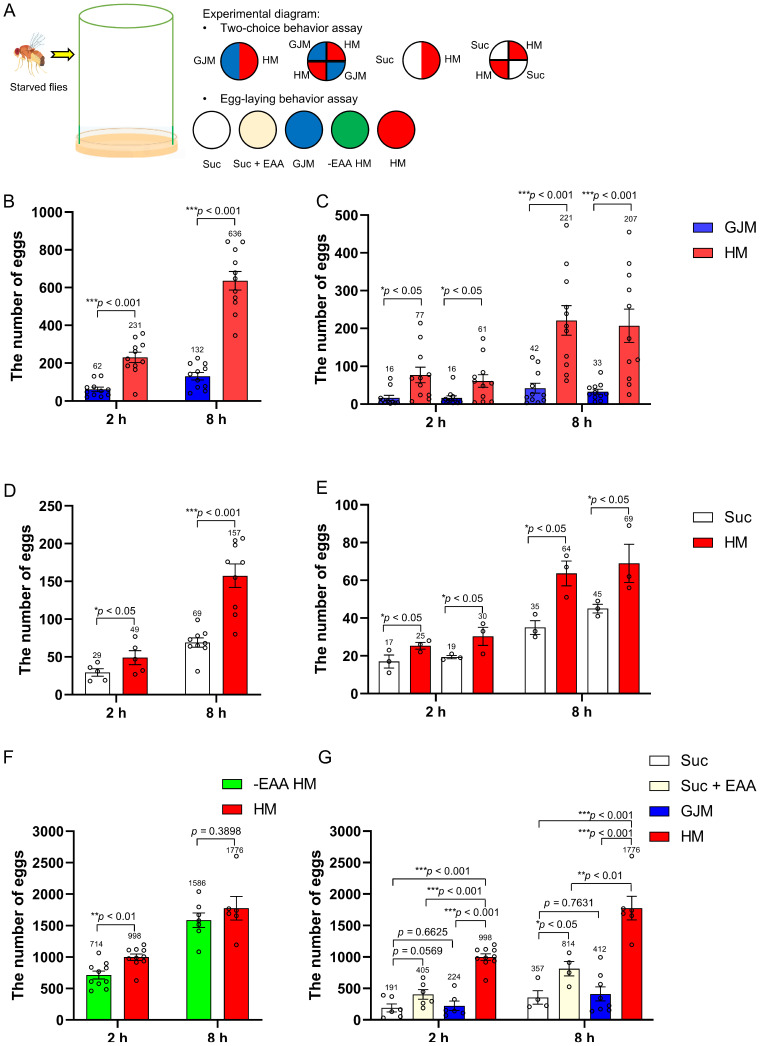
Female flies discriminate oviposition substrates and prefer HM. (**A**) Experimental schematic. (**B**,**C**) Two-choice assay: GJM vs. HM (100 starved WT females + 100 starved WT males per container). (**D**,**E**) Two-choice assay: Suc vs. HM (100 starved WT females + 100 starved WT males per container). (**F**,**G**) Eggs laid on the indicated substrates at 2 h and 8 h (150 starved WT females + 150 starved WT males per container). Suc, sucrose plate; Suc + EAA, Suc supplemented with EAAs (9.737 g/L; HM recipe as per Piper et al. [10]); -EAA HM, HM without EAAs (other components unchanged [10]). *n* ≥ 5 independent experiments; each point represents one experiment. Mean ± SEM. One-way ANOVA and two-tailed unpaired *t* test. * *p* < 0.05; ** *p* < 0.01; *** *p* < 0.001.

A hollow transparent cylinder (80 mm height, 120 mm diameter) was used as the oviposition assay device, with egg-laying culture dishes placed at the bottom, to evaluate *D. melanogaster* oviposition behavior on various substrates (Figure 1A). To examine potential differences in egg-laying preferences across different culture media, 3–5-day-old adult flies were subjected to protein deprivation prior to the oviposition tests, as amino acid sensitivity has enhanced previously [10,14]. Female flies preferentially selected the HM-enriched side (rich in amino acids) for oviposition over the GJM side (Figure 1B,C and Figure S1A–C), consistent with previous findings. In comparisons between HM and GJM, starved females preferentially chose the HM side over the sucrose-containing side (Figure 1D,E). Given the innate response that female *D. melanogaster* assess suitable environments prior to making final egg deposition decisions, the choice of substrate directly influences the number of eggs. Consequently, the greater preference for the HM-enriched diet resulted in increased egg deposition on HM (Figure 1B–E).

In the two-choice setup, females deposited significantly more eggs on HM than on GJM (HM/GJM = 4.02 at 2 h and 5.27 at 8 h). Since two-choice deposition reflects both substrate preference and egg-laying output, total egg deposition was subsequently quantified in no-choice assays, where flies were provided a single substrate per container. Under these conditions, the omission of all 10 EAAs reduced egg numbers after 2 h (Figure 1F), whereas the 8 h difference was not statistically significant. In a separate no-choice experiment, flies subjected to overnight protein deprivation deposited approximately 4–5 times more eggs on HM than those on either GJM or Suc (Figure 1G). Specifically, at 2 h, the mean fold changes for HM relative to GJM and Suc were 4.34 and 5.23, respectively; at 8 h, the corresponding mean fold changes were 4.31 and 4.97. No significant egg-laying differences were observed between GJM and Suc plates, indicating that these substrates are interchangeable as controls in this assay context.

Consistent with Figure 1F, the addition of EAAs significantly promoted oviposition behavior. Supplementary Data indicated normal ovarian development in 3–5-day-old female flies (Figure S1D). Some females on GJM retained mature eggs in the ovipositor without deposition. Eggs on HM developed normally, similar to those on a normal diet (Figure S2), confirming the suitability of HM as an oviposition substrate. Since females prefer depositing eggs on soft substrates [3]. By adjusting the acidic pH and agar content to 1% *w*/*v*, the HM recipe was optimized to maximize egg production within hours. Practically, for sterile fly preparation, the egg-collection time was reduced by at least 50% without compromising egg yield.

### 2.2. Lysine Serves as the Key Nutrient Stimulating Acute Egg Laying

Compared to GJM and -EAA HM (defined as HM lacking EAAs), HM is enriched in amino acids. To investigate the nutritional components in HM that stimulate egg laying within short timeframes, different oviposition substrates were prepared by selectively removing various nutrients. Unlike EAAs, other nutrients, such as vitamins and cholesterol, did not exhibit consistent effects on egg deposition. EAA deficiency resulted in a significant decrease in egg production within 2 h periods (Figure 1F). Furthermore, supplementing EAAs in sucrose significantly increased egg numbers compared to sucrose alone (Figure 1G), with average fold changes of 2.12 at 2 h and 2.28 at 8 h. These results confirm the findings from deletion experiments in Figure 1F and are consistent with previous studies indicating that EAA is the first available resource to which the flies exhibited strong responses.

**Figure 2:**
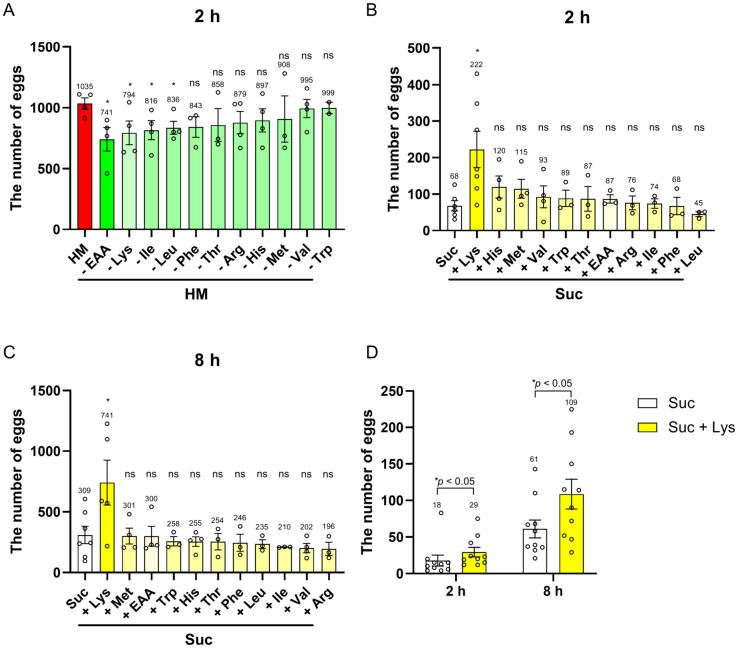
Lysine is a key EAA that promotes egg laying over short timescales. (**A**) Egg production on HM and HM lacking individual EAAs at 2 h (150 starved WT females + 150 starved WT males per container). (**B**,**C**) Egg production on sucrose plates (Suc) supplemented with 0.2% of an individual EAA at 2 h and 8 h, respectively. (**D**) Two-choice oviposition assay comparing Suc vs. Suc + Lys. Except for amino acids, all other HM components were unchanged. (**B**–**D**) A total of 100 starved WT females + 100 starved WT males per container. In panel (**A**), red indicates complete HM, dark green indicates -EAA HM, and light green indicates -EAA HM supplemented with individual essential amino acids; in panels (**B**,**C**), white indicates Suc, bright yellow indicates Suc + Lys, and pale yellow indicates Suc supplemented with other indicated amino acids or EAA mixture. Data from ≥ 3 independent experiments (*n* ≥ 3); each point represents one experiment. Statistics: one-way ANOVA and two-tailed unpaired *t* test. Data are mean ± SEM. ns, *p* > 0.05; * *p* < 0.05.

The above findings demonstrate that *D. melanogaster* actively selects environments enriched with EAAs for oviposition, suggesting that EAAs are primary nutritional factors influencing egg deposition. To identify the most relevant EAA and determine the specific EAA in HM that plays a critical role in modulating egg-laying, egg counts were performed in various EAA-omitting HMs. Significant effects on oviposition decision were observed in the absence of EAAs or lysine, reflecting a short-term reproductive phenotype (Figure 2A).

Among the 10 EAAs tested, lysine exhibited the most pronounced effect (Figure 2B,C). EAAs enhanced short-term reproductive phenotypes, with the presence of lysine resulting in an average fold change 3.26 times higher than sucrose, and a maximum variation of 6.42-fold. Lysine’s most substantial egg-laying-promoting effect corresponded with the results presented in Figure 2A. Moreover, lysine-containing cultures significantly attracted *D. melanogaster* (Figure 2D), consistent with the laying preference for female HM observed in Figure 1B–E. When lysine replaced the other 19 amino acids to generate a modified HM, this phenotype was largely reproducible, suggesting that lysine can functionally replace other amino acids during short-term exposure. Collectively, these findings support lysine as a critical EAA driving the short-term egg-laying phenotype observed in our assay.

### 2.3. Gut Transcriptomics Reveals HM-Responsive Metabolic and Endocrine Reprogramming

Consistent with the rapid reduction in egg laying behavior after acute omission of EAAs or lysine (Figure 1 and Figure 2), these nutrients in HM appear to promote oviposition on a short timescale. Oviposition-related decision-making is dynamically shaped by both internal physiological state and prior experience [24]. We therefore hypothesized that EAA/lysine intake triggers gut molecular programs that support rapid transitions in egg-laying behavior.

**Figure 3:**
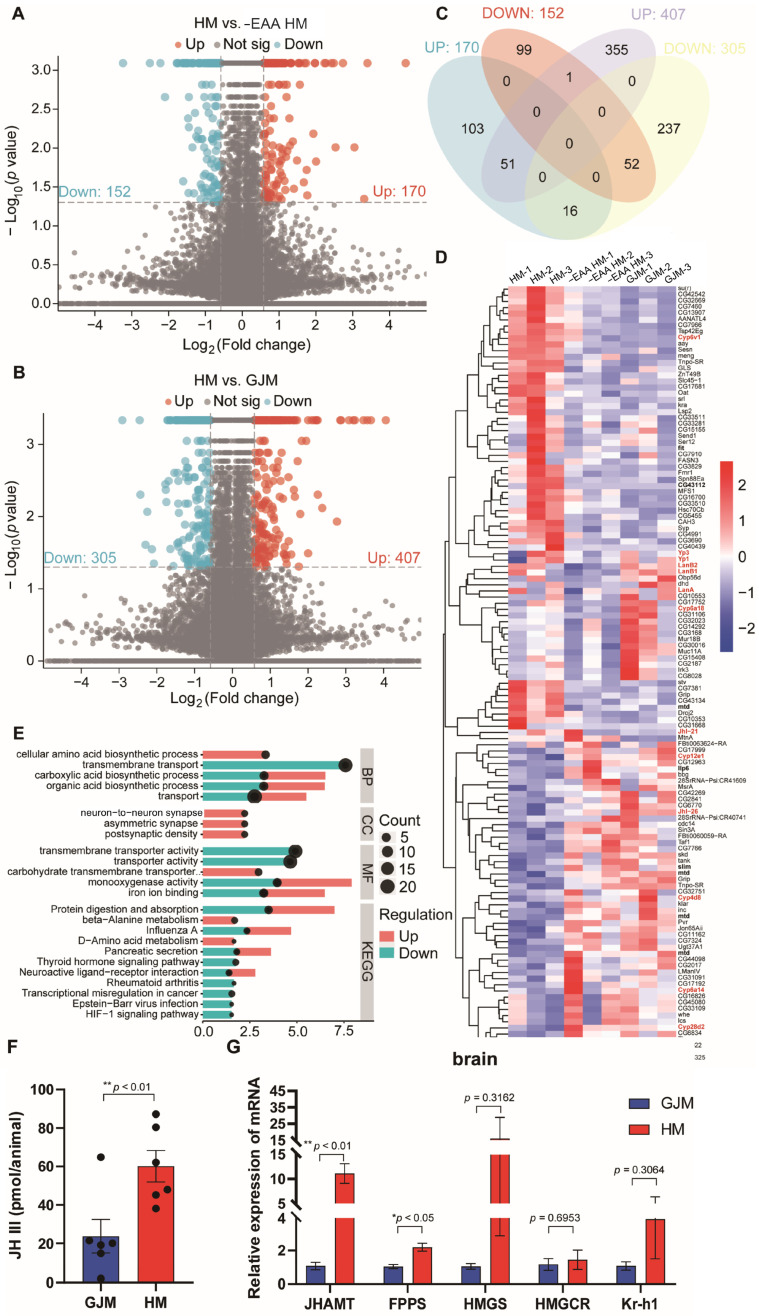
Gut transcriptomics nominates endocrine- and JH-related candidates and supports elevated JH signaling under HM. (**A**) Volcano plot of gut transcripts (40,411 total) from females that laid eggs on HM versus -EAA HM (-EAA HM, HM lacking all EAAs). Thresholds: *p* < 0.05 and fold change ≥ 1.5. Red, upregulated in HM; blue, downregulated in HM. (**B**) Volcano plot of gut transcripts (40,411 total) from females that laid eggs on HM versus GJM. Red, upregulated in HM; blue, downregulated in HM. (**C**) Venn diagram showing 51 shared upregulated and 52 shared downregulated transcripts in HM across comparisons. (**D**) Heatmap of 120 dysregulated transcripts across three groups; transcripts related to the JH signaling pathway are highlighted in red. (**E**) GO and KEGG enrichment of 98 dysregulated genes. (**F**) JH titers in females after laying on GJM or HM for 8 h; values were normalized to the number of females (GJM, *n* = 564 total; HM, *n* = 524 total) across 6 experiments (*n* = 6). (**G**) qPCR analysis of JH biosynthesis–related genes in adult brains (including the corpora allata). For each sample, 50 female brains were collected after laying on GJM or HM for 8 h; egg-laying assays were performed as described above. Expression was normalized to GJM using actin as the internal control (*n* = 3 experiments). Statistics: two-tailed unpaired *t* test. Data are mean ± SEM. * *p* < 0.05; ** *p* < 0.01.

To identify intestinal genes responsive to HM nutrients, we performed RNA-seq on gut tissues from flies maintained in GJM, -EAA HM (defined HM lacking EAAs), or HM (Figures S3A and S4A). In total, 40,411 transcripts were detected (Figure 3A,B). At the transcript level, 1860 transcripts (4.7%) were differentially expressed in HM versus -EAA HM, whereas 3121 transcripts (7.7%) were differentially expressed in HM versus GJM (adjusted *p* < 0.05). Using a more stringent cutoff (adjusted *p* < 0.05 and fold change ≥ 1.5), 314 and 700 transcripts were retained in the HM versus -EAA HM and HM versus GJM comparisons, respectively (Figure 3C). At the gene level, these contrasts contained 1776 and 2812 significant genes, respectively (adjusted *p* < 0.05), including 282 and 597 genes that also met the 1.5-fold cutoff.

**Figure 4:**
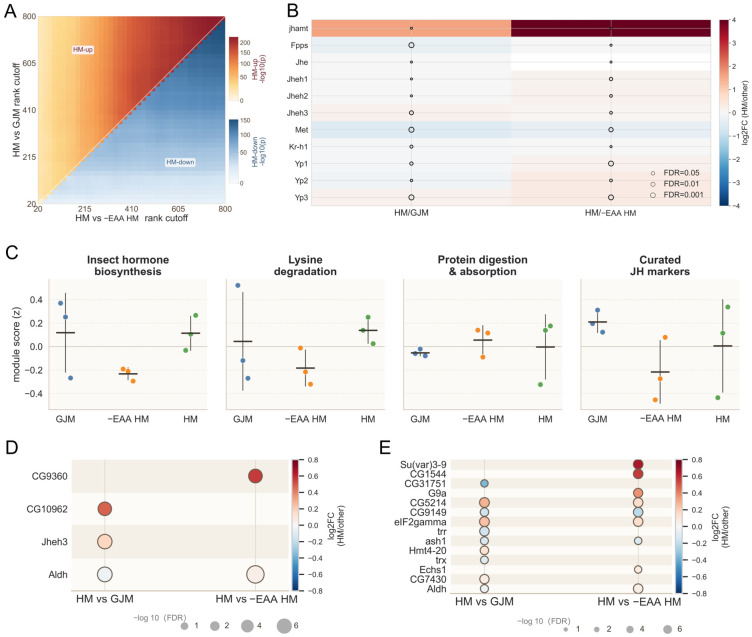
Gut transcriptomics identifies HM-responsive programs consistent with a JH-centered endocrine model of acute egg laying. (**A**) Bidirectional rank–rank hypergeometric overlap (RRHO) analysis comparing the HM vs. GJM and HM vs. -EAA HM contrasts. Warm colors indicate concordant HM-up programs, and cool colors indicate concordant HM-down programs. Color intensity represents overlap significance as −log10(*P*) from the hypergeometric test. (**B**) Heatmap of core JH-related genes across the two contrasts (HM/GJM and HM/-EAA HM). Color indicates log2FC (HM/other), and dot size indicates significance (−log10(FDR)). (**C**) Module-score analysis for selected gene sets, including insect hormone biosynthesis, lysine-related genes, protein digestion/absorption, and curated JH markers. Dots represent individual biological replicates, and black bars indicate group mean with dispersion. (**D**) Bubble plot of significantly changed genes in the KEGG pathway ko00981 (insect hormone biosynthesis). Only genes with FDR < 0.05 in at least one contrast are shown. Bubble color indicates log2FC (HM/other), and bubble size indicates −log10(FDR). (**E**) Bubble plot of genes assigned to KEGG ko00310 (lysine-related gene set). Only genes with FDR < 0.05 in at least one contrast are shown. Bubble color indicates log2FC (HM/other), and bubble size indicates −log10(FDR). Abbreviations: GJM, grape/apple juice media; HM, holidic medium; -EAA HM, essential amino acid-depleted holidic medium.

Unsupervised quality-control analyses supported the robustness of the dataset. PCA separated the three dietary conditions, whereas sample-to-sample correlation analysis showed high reproducibility among biological replicates (Figure S4B,C). Transcriptome-wide comparison further revealed broad concordance between the two HM-centered contrasts. Bidirectional RRHO analysis showed strong overlap between HM-upregulated and HM-downregulated programs (Figure 4A). Examination of a curated set of core JH-related genes highlighted coordinated changes in JH synthesis, catabolism, signaling, and response markers across the two contrasts (Figure 4B). Module-level analysis further indicated HM-associated shifts in insect hormone biosynthesis, lysine-related genes, protein digestion and absorption, and curated JH-marker programs (Figure 4C). Consistent with this pattern, gene-level visualization of KEGG ko00981 and ko00310 showed significant changes in hormone-biosynthesis-associated genes and a lysine-related gene set, respectively (Figure 4D,E). Together, these data indicate that HM elicits a reproducible gut transcriptional response consistent with coordinated metabolic and endocrine remodeling, supporting a JH-centered endocrine model for the acute promotion of egg laying.

### 2.4. Nominating Endocrine- and JH-Related Candidates for Functional Validation

HM significantly altered the expression levels of CYP6A14, CYP6A18, CYP4D8, CYP6V1, CYP12E1, and CYP28D2 (Figure 3D). Cytochrome P450 (CYP) enzymes constitute a large family of heme-containing enzymes present in animals, plants, microbes, and humans. These enzymes exhibit a variety of biocatalytic activities, including oxidation, epoxidation, hydroxylation, and demethylation. They are essential for processes such as drug metabolism, cholesterol and steroid hormone synthesis, and the metabolism of lipid-soluble vitamins. Specifically, CYP enzymes catalyze the conversion of cholesterol into active ecdysteroids, such as 20E, through complex biochemical pathways [25,26]. Christesen et al. demonstrated that the knockout of CYP6U1 resulted in decreased levels of 20E and JH titers [27]. The study also reported the enrichment of CYP6V1 in the CA, although its role in hormone synthesis remains undefined.

Yolk protein (Yp) gene expression is regulated by JH signaling. Krüppel homolog 1 (*Kr-h1*) serves as a primary JH response gene, and both *Kr-h1* and *Yp* genes have been reported to be induced by JH [15,19,28,29]. Beyond CYPs, transcriptome sequencing revealed DEGs associated with JH signaling pathways (Figure 3D). Notably, *Yp1* and *Yp3* were upregulated in HM, suggesting that JH levels vary in response to different amino acid concentrations in food sources. As previously stated, JH levels may be induced by HM. LC-MS/MS analysis showed a significant increase in JH levels (2.54-fold change) (Figure 3F), supporting this hypothesis. To further validate this finding, we observed that JH synthesis genes *JHAMT* and *FPPS* were upregulated in the brains of the HM group (Figure 3G). However, no statistically significant differences in *Kr-h1* expression were observed between GJM and HM groups, likely due to substantial variability (large error bars).

Previous studies have demonstrated that a reduction in amino acid transporters in the fat body leads to decreased egg-laying rates by regulating germline stem cell division and ovulation [30]. Transcriptome sequencing revealed significant changes in the expression levels of 15 genes encoding amino acid transporters, including slimfast, CG16700, and CG4991, all of which were downregulated in the -EAA HM and GJM groups (Figure 3D,E). It is hypothesized that the downregulation of these transporters is a consequence of amino acid deficiency and may contribute to the reduced egg-laying output. Amino acids in the fat body can influence reproduction via the target of rapamycin (TOR) signaling pathway. However, feeding rapamycin (Rap, a TOR inhibitor) did not affect egg-laying across different oviposition substrates within 8 h (Figure S3B,C). These findings suggest that TOR signaling is not a major factor in the acute egg-laying-promoting effects of EAAs or lysine.

Key RNA-seq trends were validated through qPCR (Figure S3D). While gut transcriptomics revealed diet-dependent regulation of nutrient-responsive genes, including *fit* and *Dilp6* (Figure S3E–G), the HM-induced acute egg-laying phenotype persisted in both Fit knockout (Fit^81^) and Dilp6 mutant (Dilp6^41^) flies (Figure S3H,I), indicating that these factors are not essential for the acute response under the given assay conditions. Collectively, these data demonstrate that HM induces rapid, EAA-dependent transcriptional remodeling in the gut and suggest potential endocrine/JH-related candidates, which are further functionally tested in subsequent sections.

Unsupervised analyses of gut RNA-seq profiles confirmed strong within-condition reproducibility and clear separation of HM, -EAA HM, and GJM samples (Figure S4B,C). Using a threshold-free rank–rank hypergeometric overlap (RRHO) framework, highly concordant HM-up and HM-down programs were observed across both contrasts (HM vs. GJM and HM vs. -EAA HM), supporting a robust HM-responsive signature that is independent of an arbitrary DEG cutoff (Figure 4A,B). In this context, pathway/module scoring, along with curated endocrine/JH gene summaries, revealed concomitant shifts in amino acid handling (including lysine-related metabolism) and hormone-related signatures under HM, providing orthogonal transcriptomic evidence for the endocrine framework, which is functionally tested in subsequent experiments, without implying causality (Figure 4C–E).

### 2.5. Lysine Elevates 20E and JH and Induces ETH Expression During Acute Egg Laying

**Figure 5:**
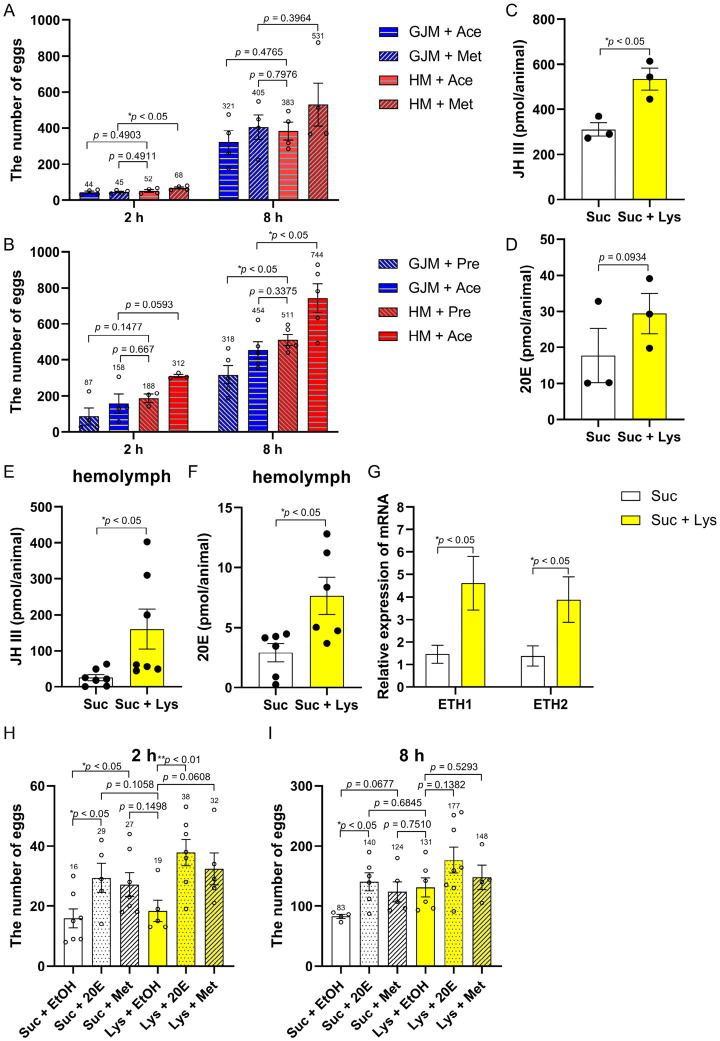
Lysine modulates 20E, ETH, and JH levels to promote short-term egg laying in *D. melanogaster*. (**A**) Eggs laid on GJM and HM by flies treated with the JH analog methoprene (Met) over 2 h and 8 h (*n* = 4 experiments; each point represents one experiment). (**B**) Eggs laid on GJM and HM by flies treated with the JH antagonist precocene (Pre) over 2 h and 8 h (*n* = 5 experiments; each point represents one experiment). For (**A**,**B**), acetone (Ace) served as the vehicle control; Met or Pre was topically applied in acetone to the ventral side. (**C**,**D**) Whole-body JH and 20E titers in females after 8 h oviposition on the indicated substrates (150 females + 150 males per group; *n* = 3 experiments; each point represents one experiment). (**E**,**F**) Hemolymph JH and 20E levels in females after 8 h oviposition on the indicated substrates (200 females + 200 males per group; *n* ≥ 6 experiments; each point represents one experiment); hemolymph was collected from females after laying. (**G**) ETH expression in females after 8 h egg-laying assays on sucrose- or lysine-containing media (50 starved WT females + 50 males per container; *n* = 5 experiments); expression was normalized to Suc using actin as the internal control. (**H**,**I**) Eggs laid on the indicated media by flies treated with a 20E analog and/or Met over 2 h and 8 h. EtOH served as the vehicle control; Met and/or 20E in EtOH was topically applied to the ventral side. Each point represents 50 starved WT females + 50 males; *n* = 6–7 experiments. Statistics: two-tailed unpaired *t* test. Data are mean ± SEM. * *p* < 0.05; ** *p* < 0.01.

In adults, JH is a gonadotropic hormone that promotes reproductive development and ovulation in *Drosophila* [15,31]. Consistent with the role of JH in the acute assays, gut transcriptomics revealed differential expression of JH pathway-related genes under amino acid–rich conditions (Figure 3D), and LC-MS/MS combined with qPCR confirmed elevated JH titers and increased expression of JH biosynthesis genes under HM (Figure 3F,G). Consequently, we tested whether modulation of JH signaling could alter egg-laying output over short time intervals. Topical application of the JH analog methoprene (Met) increased egg deposition on GJM (Figure 5A and Figure S5A), whereas the JH antagonist precocene (Pre) reduced egg deposition on HM, returning it to levels not significantly different from untreated GJM controls (Figure 5B and Figure S5B). Collectively, these perturbations are consistent with the involvement of JH signaling in the HM-induced acute increase in egg-laying.

Given the strong lysine dependence of the acute egg-laying phenotype (Figure 2), we next investigated whether lysine influences endocrine hormone titers. Compared to sucrose alone, females maintained on sucrose supplemented with lysine exhibited significantly higher whole-body JH and 20E titers, with increases of 1.72-fold and 1.66-fold, respectively (Figure 5C,D). In hemolymph collected from egg-laying females, lysine supplementation resulted in 6.25-fold and 2.62-fold higher JH and 20E levels than in sucrose controls, respectively (Figure 5E,F). Collectively, these data indicate that lysine intake elevates both circulating and whole-body JH and 20E levels during the acute assay.

ETH synthesis has been reported downstream of ecdysone signaling, suggesting that 20E can regulate ETH expression. Meiselman et al. administered 20E injections to adult *D. melanogaster* and observed increased transcription of ETH and ETH receptor (ETHR) 4 h later [19], consistent with a steroid-dependent induction of ETH. Given that ETH functions as an allatotropin to stimulate JH biosynthesis [16,17,18,19,20,21,22,23], we subsequently investigated whether lysine modulates this 20E–ETH–JH cascade. Lysine indeed increased ETH transcript levels (Figure 5G), along with causing elevated 20E and JH titers (Figure 5C–F). Furthermore, a 20E analog promoted egg-laying on sucrose media, yielding no significant difference compared to lysine-supplemented media (Figure 5H,I). In conjunction with the methoprene/precocene perturbations, these findings support a model in which lysine promotes acute egg-laying via a 20E–ETH–JH endocrine cascade.

### 2.6. JH Promotes Egg Laying and Is Associated with Increased ECM-Related Gene Expression in the Ovarian Muscle

JH signaling promotes ovulation in adult *Drosophila*, with the ovarian muscle serving as a key target tissue in which JH induces the expression of ECM-related genes, including laminin-related genes, necessary for efficient egg release [15]. Consequently, we investigated whether JH activation in our acute assays correlates with an upregulation of ECM-related gene expression.

Consistent with the observed egg-laying phenotypes, methoprene enhanced egg deposition, whereas precocene inhibited it (Figure 5A,B). To evaluate the transcriptional effects of JH signaling under these conditions, females were treated with methoprene (Met) or precocene (Pre), and tissues were collected following an 8 h egg-laying assay for subsequent qPCR analysis. *Kr-h1*, a primary JH response gene whose transcription is directly induced by JH, was used as a marker for JH signaling activity [31]. *Kr-h1* expression was upregulated by Met and downregulated by Pre, demonstrating that these treatments effectively modulate JH signaling in vivo.

**Figure 6:**
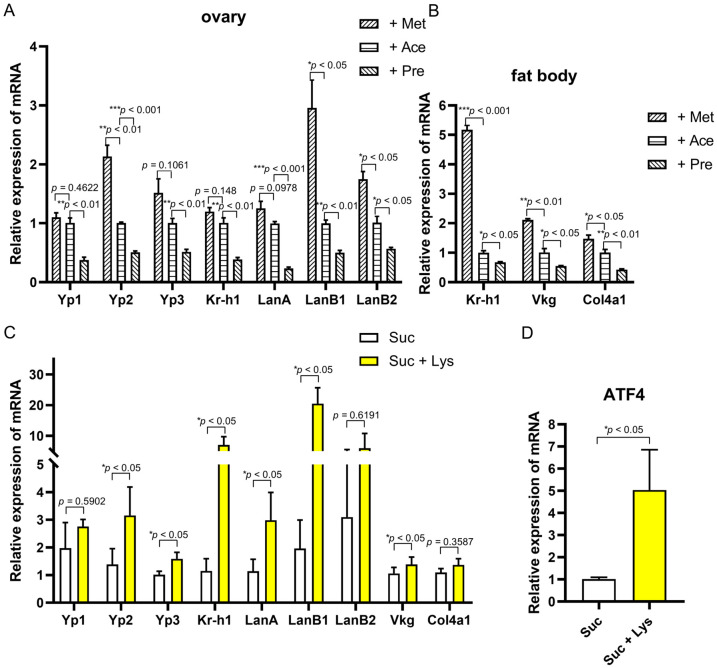
JH promotes ovulation in *D. melanogaster* by inducing ECM-related gene expression. (**A**) Gene expression in ovarian tissue from egg-laying females after 8 h feeding with a JH analog. (**B**) qPCR analysis of collagen genes in the fat body of egg-laying females on the indicated media after 8 h treatment with JH mimetics. For (**A**,**B**), acetone (Ace) served as the vehicle control; methoprene, Met; precocene, Pre; each vial contained 20 WT females and 5 males; *n* = 3 experiments. qPCR signals were normalized to Ace using Rp49 as the internal reference gene. (**C**) Expression of ECM-related genes in females after 8 h egg-laying assays (50 starved WT females + 50 males per container). (**D**) Hemolymph ATF4 levels in females after 8 h egg-laying assays on Suc or Suc + Lys; ATF4 was measured in hemolymph collected after laying. For (**C**,**D**), expression was normalized to Suc using actin as the internal control. Data are mean ± SEM from ≥ 3 independent experiments (*n* ≥ 3). Statistics: unpaired *t* test. * *p* < 0.05; ** *p* < 0.01; *** *p* < 0.001.

Methoprene treatment significantly upregulated ECM-related gene expression, whereas precocene treatment suppressed these transcripts (Figure 6A,B), consistent with previous findings [15]. Notably, these transcriptional changes in ECM genes aligned with the observed egg-laying phenotypes under methoprene and precocene treatments (Figure 5A,B), providing further evidence of a relationship between JH signaling and ECM gene regulation in reproductive tissues, including the ovarian muscle layer, during acute egg laying. Additionally, lysine supplementation was associated with increased expression of both JH signaling markers and ECM-related genes in ovarian tissue, including the ovarian muscle (Figure 6C), suggesting activation of JH signaling downstream of lysine intake.

### 2.7. Lysine Is Associated with ATF4 Induction and No Detectable Change in Octopamine Levels During Acute Egg Laying

Hemolymph from egg-laying females fed sucrose supplemented with lysine exhibited significantly higher ATF4 levels compared to sucrose-only controls (Figure 6D). ATF4 is a key downstream effector of the amino acid sensor GCN2 within the integrated stress response (ISR) and has been implicated in regulating CNMa and ETH expression [32]. This finding suggests a potential mechanism by which amino acid availability may influence ETH signaling in the context of our acute assay.

ETH has been shown to influence ovulation through octopaminergic circuits [19,33]. However, no significant differences in OA content were observed in the heads, ovaries, or hemolymph across conditions (Figure S6), indicating that OA levels are not substantially altered in this short-term phenotype. Given that ETH regulates JH biosynthesis, lysine-induced ETH expression may contribute to elevated JH titers and ECM-related gene expression (Figure 5 and Figure 6), thereby facilitating acute egg laying.

## 3. Discussion

**Figure 7:**
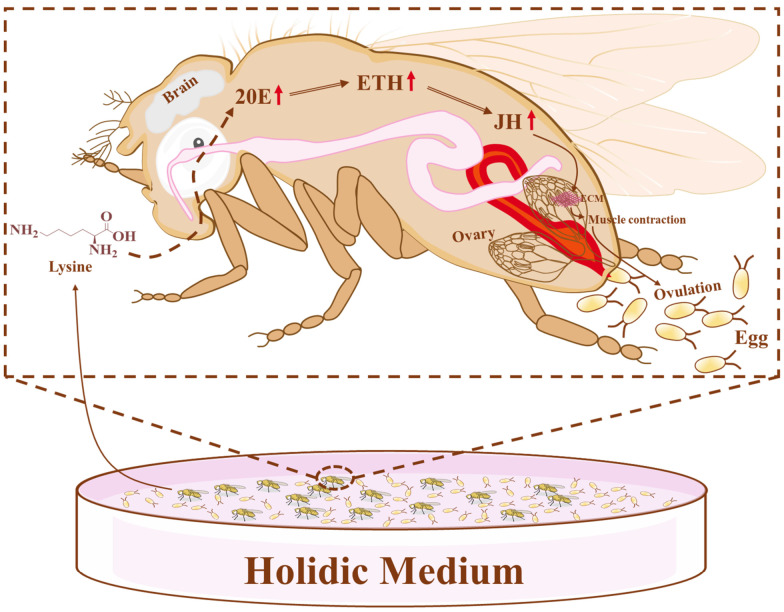
Model for lysine-driven regulation of short-term egg-laying behavior in *D. melanogaster*. Mated flies sense dietary nutrients (e.g., sugars, proteins, lipids, vitamins, and metal ions), which elicit broad changes in gene expression. Amino acid intake—represented here by lysine—increases the levels of 20E, ETH, and JH. These hormones act on the ovaries to regulate ovulation, leading to altered short-term reproductive output (phenotype observed in this study). The red arrows indicate increased levels of the corresponding substances.

Reproduction is closely linked to nutrient availability; however, the specific signals that translate nutrients into rapid egg-laying output have remained challenging to elucidate using conventional, nutritionally undefined egg collection media. Utilizing a holidic medium and an oviposition assay that compares substrates within the same chamber, we identify lysine as a key acute cue that stimulates egg laying within hours. The data support a model in which lysine elevates 20E levels, induces ETH expression, and enhances JH signaling, concomitant with increased expression of ECM-related genes in the ovarian muscle (Figure 7).

Amino acids are well-established long-term determinants of *Drosophila* fecundity [8,12,13]. By focusing on a short time window (2–8 h), we differentiate rapid egg deposition from the slower effects on oogenesis and demonstrate that EAAs—particularly lysine—can influence egg-laying output within the timescale of behavioral decision-making. Furthermore, HM increases female residence time and preference relative to juice-based media (Figure 1 and Figure S1), indicating that nutrient-dependent choice and endocrine regulation of the reproductive tract may function synergistically to modulate acute egg-laying output.

Lysine likely modulates endocrine regulation through its influence on ETH, a conserved allatotropin that promotes JH biosynthesis, with 20E acting upstream of ETH in several insect species [16,17,18,19,20,21,22,23]. In our assays, lysine supplementation increased both whole-body and hemolymph 20E and JH titers and induced ETH transcription (Figure 5). Consistently, methoprene enhanced egg laying on GJM, while precocene suppressed the HM-induced phenotype (Figure 5A,B), supporting the involvement of JH signaling in acute egg-laying output. A 20E analog similarly increased egg laying (Figure 5H,I), further implicating steroid signaling in this cascade. Collectively, these findings establish a link between a single dietary amino acid and a 20E–ETH–JH axis that operates on a timescale of hours. Notably, lysine has also been associated with reproductive performance in other taxa [34,35,36,37,38,39,40], suggesting it may serve as a broadly utilized reproductive nutrient cue.

Downstream of JH, the ovarian muscle is a recognized target tissue where JH induces ECM genes essential for efficient ovulation [15]. Consistent with this framework, methoprene and precocene bidirectionally regulate *Kr-h1* and ECM-related transcripts, while lysine is associated with elevated expression of JH signaling components and ECM genes (Figure 6). Although muscle contractility was not directly measured, these transcriptional changes establish a mechanistic link between endocrine activation and the egg-laying phenotype.

Several upstream nutrient-sensing pathways may link lysine to this hormonal cascade. Transcriptomic analysis reveals rapid metabolic reprogramming in the gut under high-amino acid conditions, including alterations in amino acid transporters, hormone-related genes, and nutrient-responsive factors (e.g., fit and Dilp6), as well as changes in diet-associated transcripts (e.g., MsrA and mtd/OXR1) (Figure 3 and Figure 6), consistent with prior studies of these nutrient-responsive factors [41,42,43,44]. Additionally, induction of ATF4 in the hemolymph under oviposition-promoting conditions (Figure 6D) is consistent with activation of the GCN2–ATF4 integrated stress response, which has been implicated in fat-body regulation of reproductive neuropeptides, including ETH [32]. Beyond internal nutrient sensing, amino acid-responsive taste circuits can influence egg laying [10], and a sensory contribution to the lysine-dependent preference observed remains plausible. Beyond internal nutrient sensing, peripheral amino acid taste pathways may also contribute to the acute oviposition response. Amino acids can be detected by taste neurons on the legs and labellum, and Ir76b-, Ir25a-, and IR94e-associated pathways have been implicated in amino acid sensing, feeding, and egg-laying behavior [10,45,46]. In particular, IR94e-expressing taste cells have been shown to influence both feeding and oviposition, linking amino acid detection to egg-laying circuits. Therefore, peripheral taste sensing and the Lysine–20E–ETH–JH pathway should not be viewed as mutually exclusive. Rather, taste-mediated neural input may act upstream of, in parallel with, or together with endocrine regulation. Our transcriptomic and hormone data support an endocrine response associated with lysine supplementation, while future experiments using amino acid taste-defective flies, such as Ir76b-, Ir25a-, or Ir94e-related mutants or neuronal manipulations, will be required to define the relative contribution of peripheral taste sensing.

This study has several limitations. ETH was primarily assessed at the transcript level, and pharmacological perturbations may exert pleiotropic effects. Additionally, increased egg deposition may result from alterations in oviposition choice, residence time, and/or changes in the rate of ovulation and oviductal transit. Consequently, the 20E–ETH–JH cascade is presented as a supported model for acute regulation rather than a fully resolved causal pathway. Notably, differences in egg output across media diminish over longer time frames (36–72 h), suggesting that acute nutrient cues are integrated with longer-term physiological adaptations and experience-dependent oviposition behavior [30].

In summary, the results identify lysine as an acute nutritional cue capable of initiating endocrine signaling associated with rapid egg laying in *D. melanogaster*. In addition to providing a defined experimental platform for dissecting short-timescale nutrient–hormone interactions, this study offers a practical approach for efficient egg collection and highlights endocrine nodes that could be targeted to modulate insect fecundity.

## 4. Materials and Methods

### 4.1. Fly Strains and Husbandry

Fly stocks were maintained on a standard diet (ND: 6.0 g coarsely ground corn, 10.0 g 10% (*w*/*v*) glucose, 1.5 g instant yeast, 1.0 g 1% (*w*/*v*) agar, 100 mL water). All experiments were conducted at 25 °C under a 12:12 h light/dark cycle. The wild-type strain w^1118^ was used. Only adult female *D. melanogaster* were included in subsequent experiments. Immediately after eclosion, females and males were separately collected and maintained on ND (25 females or 50 males per vial) for 3–5 days before experimentation.

### 4.2. Preparation of Oviposition Substrates

The nutrient composition of each medium (*w*/*v*; g per 100 mL water) was as follows: the nutrient-deprivation plate contained 3.0–3.5% agar and 1.712% sucrose, because prolonged complete nutrition starvation extremely easily caused lethality before the oviposition tests; the grape juice medium (GJM) contained 1% agar, 1.2% sucrose, and 25% (*v*/*v*) fresh grape juice; and the sucrose medium (Suc, minimal oviposition medium controls according to [47]) contained 1% agar and 1.712% sucrose.

The HM, based on Piper et al. [12] with modifications, contained 1% agar, 1.712% sucrose, L-isoleucine (1.82 g/L), L-leucine (1.21 g/L), L-tyrosine (0.42 g/L), buffer, metal ions (calcium chloride, copper sulfate, iron sulfate, anhydrous magnesium sulfate, manganese chloride, zinc sulfate), and cholesterol (dissolved in ethanol). After autoclaving, the HM was supplemented with sterile stock solutions of essential amino acids (60.51 mL/L), non-essential amino acids (60.51 mL/L), sodium glutamate, vitamins, sodium folate, and nucleic acid liquid (NAL). Finally, pH was adjusted to 4.5. The -EAA HM (defined as HM lacking EAAs) was prepared identically to the HM, except that 10 EAAs (including L-isoleucine (Ile), L-leucine (Leu), L-arginine (Arg), L-histidine (His), L-lysine (Lys), L-methionine (Met), L-phenylalanine (Phe), L-threonine (Thr), L-tryptophan (Trp), and L-valine (Val)) were omitted. Following the protocol outlined by Song et al. [24], egg-laying dishes were prepared by adjusting individual amino acid at 0.1% mass fractions.

As described previously [47], the lysine-supplemented medium (Suc + Lys) contained 1% agar, 0.2% L-lysine, and 1.712% sucrose, whereas the EAA–supplemented medium (Suc + EAA) contained 1% agar, 1.712% sucrose, and 0.2% each of 10 EAAs.

All media were poured into 120 mm dishes.

### 4.3. Measurement of Oviposition in D. melanogaster Under Different Conditions

For the egg-laying performance assay, 100 or 150 pairs of 3–5-day-old adults maintained on ND were collected (1:1 female-to-male ratio) and subjected to protein deprivation on a 3.0–3.5% (*w*/*v*) agar sucrose plate for 16 h (overnight). As described previously [3,8,10], the flies were then transferred to the indicated oviposition media and maintained at 25 °C and 60% relative humidity. Egg counts were recorded after 2, 8, and 24 h to assess the reproductive output of protein-deprived flies on various substrates.

For the oviposition preference assay, 100 females and 100 males were starved for 16 h (overnight protein deprivation) and then placed on plates containing two oviposition substrates. Egg distribution on each substrate was photographed and recorded at 2, 8, and 24 h. All assays were performed at 10:00 am using a transparent hollow cylinder (80 mm height, 120 mm diameter) as the oviposition chamber.

### 4.4. RNA Sequencing, Data Analysis, and Quantitative RT-PCR

Female flies that laid eggs on HM, -EAA HM (defined HM lacking EAAs), or GJM were dissected to obtain gut tissue (20 flies per sample; three biological replicates). Total RNA was extracted using MagZol™ reagent (TRIzol, Invitrogen, Carlsbad, CA, USA), treated with DNase, and used for cDNA synthesis and library construction (VAHTS^®^ Universal V8 RNA-seq Kit (Vazyme, Nanjing, China)). Libraries were sequenced on an Illumina HiSeq X Ten platform (San Diego, CA, USA). Raw reads were trimmed and filtered, aligned to the *Drosophila* reference genome, and transcript abundance was quantified as FPKM. Differentially expressed genes (DEGs) were identified using fold change ≥ 1.5 and *p* < 0.05. Furthermore, to limit dependence on a single DEG threshold, rank–rank hypergeometric overlap (RRHO) was applied to assess transcriptome-wide ranking concordance for HM vs. GJM and HM vs. -EAA HM. Genes were ranked by signed significance: log2FC(HM/other) × −log10(FDR). To ensure a consistent direction (log2FC[HM/other]), fold-change signs were inverted during plotting when required. Additional summaries included curated endocrine/JH gene maps, targeted KEGG pathway views (including insect hormone biosynthesis and lysine degradation), and sample-level module scoring. A heatmap was generated using https://www.bioinformatics.com.cn, and GO/KEGG enrichment analyses were performed after Bonferroni correction, excluding terms with adjusted *p* > 0.05. Bioinformatic analyses were conducted using OmicShare and related online tools.

For quantitative PCR, RNA was extracted from the gut, head, ovary, fat body, ring gland (50 individuals per tissue), and hemolymph (50 individuals) using TRIzol reagent (Invitrogen) and reverse-transcribed with the FastKing RT Kit (KR116-02, Tiangen, Beijing, China). Quantitative PCR was performed using SYBR qPCR Master Mix (BL697A, Biosharp, Hefei, China) on an LC96 system (LC96, Roche, Basel, Switzerland), with results averaged across three replicates. Relative expression was calculated using the 2^−ΔΔCt^ method and normalized to Rp49 or actin. Primer sequences are provided in Supplementary Table S1 and were designed based on previous studies [15,19,28,32,41].

### 4.5. Tissue Dissection and Observation

Heads and ovaries were dissected separately in ice-cold phosphate-buffered saline (PBS). Tissue morphology was examined under a stereomicroscope (10× magnification). For isolation, tissues were transferred to a 1.5 mL microcentrifuge tube and centrifuged at 12,000 rpm for 10 min at 4 °C.

### 4.6. Hemolymph Collection

For hemolymph extraction, 50 female flies were decapitated and placed into a punctured 0.5 mL microcentrifuge tube containing a glass–wool filter. This tube was inserted into a 1.5 mL microcentrifuge tube and centrifuged at 12,000 rpm for 10 min at 4 °C. Each LC–MS/MS sample consisted of 200 μL hemolymph.

### 4.7. LC-MS/MS

Female flies that laid eggs for 8 h on different media were collected and processed as described [48,49]. Whole bodies were homogenized in 400 μL methanol, then centrifuged at 12,000 rpm for 10 min, and the supernatant was transferred to 300 μL hexane. After three sequential centrifugations at 3000 rpm, the hexane phase was dried, resuspended in 100 μL methanol, sonicated, centrifuged twice at 14,000× *g*, and stored at −80 °C. Hemolymph (200 μL) was homogenized with 100 μL methanol, centrifuged, and stored at −80 °C.

Standard dilutions of JH III, 20E (1–100 ng/mL), and octopamine (OA, 2.5–1000 ng/mL) were prepared in methanol. Chromatographic separation was performed on a Waters ACQUITY UPLC BEH C18 column (Milford, MA, USA) (50 mm × 2.1 mm, 7 μm) using the following gradient: 0–2 min, 100% A; 2–4 min, 50% A; 4–7 min, 0% A; 7–9 min, 100% B; 9–9.1 min, 0–100% A; 9.1–10 min, 100% A. The flow rate was 0.3 mL/min, and the injection volume was 10 μL. The retention times for OA, 20E, and JH III were 0.47, 4.64, and 6.47 min, respectively.

Mass spectrometry was performed on an AB SCIEX Triple Quad™ 6500 (Applied Biosystems, SCIEX, Shanghai, China) in positive electrospray ionization (ESI) mode using multiple reaction monitoring (MRM) with the following parameters: DP 80 V, CE 30 V, curtain gas 35, collisionally activated dissociation 8, ion-spray voltage 5500 V, and ion gas 1 and 2 at 20 and 0. Data were processed using AB SCIEX Analyst 1.6.

### 4.8. Pharmacological Perturbations

After 3–5 days of group housing, adult males and females were cold-anesthetized and topically administered hormone analogs to the ventral abdomen [15,18,19,20,49]. Methoprene (Met) or precocene II (Pre) were each diluted to 1 µg/µL, with acetone (Ace) as the vehicle control. The 20E analog was diluted to 1 µg/µL in ethanol (EtOH), with EtOH as the control. The application volume was 0.5 µL per fly. All treatments were completed within 20 min; following a brief recovery period, the flies were individually transferred to oviposition dishes for egg-laying assays.

To assess JH signaling–related gene expression, 10 µL Met or Pre in acetone was applied to the surface of ND, and adult females were transferred to the corresponding vials. After 8 h of oviposition, ovaries and fat bodies were dissected and collected for RNA extraction.

### 4.9. Statistics

Data are presented as mean ± SEM. Figures were generated using GraphPad Prism 8.0.2, R (v4.2.3), and www.xiantao.love. Comparisons were performed using one-way ANOVA and two-tailed unpaired *t*-tests. Significance was defined as: ns, *p* > 0.05; * *p* < 0.05, ** *p* < 0.01, *** *p* < 0.001.

## Data Availability

The original contributions presented in this study are included in the article/Supplementary Materials. Further inquiries can be directed to the corresponding author.

## Supplementary Materials

The following supporting information can be downloaded at: https://www.mdpi.com/article/10.3390/ijms27115065/s1 [50,51,52,53].
