# Supplementary material for: Lysine Triggers Acute Oviposition by Activating the 20E-ETH-JH Signaling Cascade in Drosophila melanogaster
Source: Int J Mol Sci. 2026 Jun 3;27(11):5065. doi: 10.3390/ijms27115065 (PMC13257574; doi:10.3390/ijms27115065)
Supplement: Supplementary file 1 [file ijms-27-05065-s001.zip › ijms-4304008-supplementary.pdf]

Supplementary Material

Lysine Triggers Acute Oviposition by Activating the 20E–ETH–JH Signaling Cascade in *Drosophila melanogaster*

Siran Yu[1, 2 #], Gang Zhou[1 #], Xiaolu Wang[1], Liming Zhang[1], Ping Li[1 *]

[1] School of Life Sciences and Technology, Tongji University, Shanghai 200092, China.

[2] College of Public Health, Shanghai University of Medicine & Health Sciences, Shanghai 201318, China.

[*] Corresponding author. E-mail address: liping01@tongji.edu.cn.

[#] These authors contributed equally to this work.

STAR Methods

Fly Stocks and Husbandry

Fly stocks were kept alive on a standard fly diet (ND, in short: 6.0 g of coarsely - ground corn (6% w/v), 10.0 g of 10% glucose, 1.5 g of instant yeast (1.5%), 1.0 g of 1% agar, along with 100 mL of water. Every single experiment was conducted at 25°C under a 12:12 - hour light/dark cycle. w^{1118} was chosen to be the wild type. This study got all data from female contributors. Soon after eclosion, we picked females and males and put them in ND vials (25 female flies per vial or 50 male flies per vial) for 3-5 days until the experiments.

Recipes for making different egg laying medium

Proportions of nutrients in each medium (weight/water volume) are as follows.

Nutrition deprivation plate: 3-3.5% agar, and 1.712% sucrose.

GJM: 1% agar, 1.2% sucrose, and 25% fresh grape juice.

Sucrose medium (Suc for short): 1% agar, and 1.712% sucrose.

HM was prepared according to the formula provided by Piper *et al.* (1) with some modifications, combining 1% agar, 1.712% sucrose, L-isoleucine (per liter: 1.82 g), L-leucine (per liter: 1.21 g), L-tyrosine (per liter: 0.42 g), buffer, metal ions (including calcium chloride, copper sulfate, iron sulfate, anhydrous magnesium sulfate, manganese chloride, zinc sulfate), and cholesterol (dissolved in EtOH). This mixture is autoclaved at 120°C for 15 minutes, cooled to 50-55°C, and then supplemented with sterilized essential amino acid stock solution (per liter: 60.51 mL, including L-arginine, L-histidine, L-lysine, L-methionine, L-phenylalanine, L-threonine, L-valine, L-tryptophan), sterilized non-essential amino acid stock solution (per liter: 60.51 mL), sodium glutamate stock solution, vitamin solution, sodium folate, and nucleic acid liquid (NAL for short). The pH is set to 4.5. Later, the addition of 20 amino acids is adjusted to 0.1 g/100 mL each.

The chemically defined medium lacking EAAs (- EAA HM for short) were prepared without L-isoleucine, L-leucine, L-arginine, L-histidine, L-lysine, L-methionine, L-phenylalanine, L-threonine, L-valine, and L-tryptophan, maintaining the same composition and preparation method as HM.

Lysine containing medium (Suc + Lys for short): 1% agar, 0.2% L-lysine, and 1.712% sucrose.

Sucrose medium with supplementary EAAs (Suc + EAA for short) incorporates 10 EAAs at 0.2% (The addition of each amino acid is equal) in the sucrose and agar solution, mixed thoroughly and autoclaved for preparation.

All the medium mentioned above were poured in 120-mm dishes.

Measurement of oviposition in *D. melanogaster* under different conditions

For egg-laying performance assay, 100 or 150 female and male fruit flies aged between 3-5 days that were ND feeding were collected, respectively. The female-to-male ratio was 1:1. They were subjected to protein deprivation treatment on a sucrose agar plate with a 3.5% (w/v) agar. After 16 hours (overnight), they were transferred to different oviposition mediums and cultured at 25°C and 60% relative humidity. The number of eggs laid within 2 hours, 8 hours, and 24 hours was counted, respectively. For oviposition preference test, 100 female and 100 male flies were collected and starved for 16 hours (overnight). After the starvation, they were transferred to plates containing two different oviposition substrates. The distribution of eggs laid on each substrate within 2 hours, 8 hours, and 24 hours was photographed and recorded, respectively. These assays were mainly carried out from 10:00 to 18:00 in a hollow transparent cylinder (80 mm height, 120 mm diameter) as the oviposition device.

RNA Sequencing, data analysis, and quantitative RT-PCR (qPCR)

Female flies that deposited their eggs on HM, - EAA HM, and GJM were cut open to acquire gut samples (20 guts made up one sample). Three replicates were set aside for each group. For fly tissue's RNA-seq, total RNA was extracted via MagZolTM Reagent (Trizol Reagent), underwent DNase treatment, cDNA synthesis, and library preparation as instructed by the manufacturer (VAHTS® Universal V8 RNA-seq Library Prep Kit for Illumina, NR605), and was sequenced on the Illumina Hiseq X Ten platform (San Diego, CA, United States). We trimmed the raw reads and then filtered them. Then, clean data were mapped onto the *Drosophila* genome, and uniquely mapped reads were gathered. The FPKM (Fragments per kilobase of exon per million fragments mapped) was employed to calculate transcript abundances.

Transcripts exhibiting a fold change of ≥ 1.5 and *p* supposing $p < 0.05$ were

acknowledged as differentially expressed. A heatmap was put together by www.oinformatics.m.n, an online platform for data analysis and visualization. The functional annotation of DEGs was estimated via GO (http://www.neontology.g) and KEGG pathway enrichment analysis (http://www.nome.p/kegg). The term would be excluded if its p-value went beyond 0.05 after carrying out the Bonferroni correction. We further used OmicShare tools and online web services (www.oinformatics.m.n and www.antao.ve) for bioinformatics analysis and data processing (2).

Regarding the qPCR mentioned before, total RNA was extracted from fly tissue (For the gut, head, ovary, and fat body, 20 parts were prepared for one sample separately. About 50 brains and 50 hemolymphs were respectively readied for one sample.) by making use of TRIzol reagent (Invitrogen, USA) following the manufacturer's rules. After that, the purified RNA products were reverse-transcribed into cDNA via the FastKing RT Kit (KR116-02, Tiangen, China). We carried out qPCR tests with the Universal SYBR qPCR Master Mix (BL697A, Biosharp, China) on a real-time PCR system (LC96, Roche, Basel, Switzerland) and obtained the data by taking the average of three replicates. The relative mRNA expression was ascertained via the ΔΔCT method, with Rp49 or actin as the endogenous gene for normalization. Referring to prior studies (3-8), the specific primers for gene amplification are noted in Supplementary Table 1.

Tissue dissection and observation

Heads and ovaries were dissected in the cold phosphate-buffered saline (PBS), respectively. The stereoscope (×10 magnification) could be used for the observation. For the isolation, issues were transferred to a 1.5 mL Eppendorf tube and subjected to centrifugation at 12,000rpm at 4°C for 10 minutes.

Hemolymph collection

For hemolymph extraction, 50 female flies were decapitated and set in a 0.5 mL tube that got punctured and has a glass wool filter. The tube was then transferred to a new 1.5 mL microcentrifuge tube. Loaded into a 5 mL Eppendorf tube and centrifuged at 12,000 rpm at 4°C for 10 minutes. One sample for LC-MS/MS had 200 hemolymph collected. For one LC-MS/MS sample, 200 hemolymph was gathered up.

LC-MS/MS

We collected female flies that spent 8 hours laying eggs on different mediums in the laying assays. The preparation of samples followed previous descriptions, with some modifications (9-11). In a brief description, whole body samples were homogenized with 400 μL methanol and then spun at 12,000 rpm for 10 minutes at 4°C. We transferred the supernatant into a new tube containing 300 μL hexane for the extraction purpose. 20 minutes after the start, we conducted centrifugation at 3,000 rpm at 4°C for 10 minutes. We performed this step three times again. The upper hexane layer got transferred into a new tube and dried in a vacuum concentrator. It was then resuspended in 100 μL of methanol and underwent sonication for 5 minutes. We centrifuged the samples twice for 10 minutes at 14,000×g at 4°C and then kept the supernatant at -80°C until analysis. Once centrifugation at 12,000 rpm at 4°C for 10 minutes is done, transfer the supernatant to a new tube, dry it in a vacuum concentrator, and store at -80 °C until analysis.

Standard dilutions (including JH III and 20E) of 100, 50, 25, 10, 5, 2.5, 1 ng/mL in methanol were used for calculation of the standard curve. As for OA, the dilutions in methanol are 1,000, 500, 250, 100, 50, 25, 10, 5, 2.5 ng/mL.

A Waters ACQUITY UPLC BEH C18 Column of size 50 × 2.1 mm. We adopted 7 μm and 130 Å for LC separation. We made 1% formic acid methanol solvent A and methanol solvent B. The details are presented as: 0-2 minutes, 100% A; 2-4 minutes, 100% A going to 50% A; 4-7 minutes, 50% A going to 0% A; 7-9 minutes, 100% B; 9-9.1 minute brings about A going from 0% to 100%, and 9.1-10 minute 100% A. With a flow rate set at 0.3 mL/min, the injection volume was 10 μL. Every sample's total run time was measured at 10 minutes. The retention time of OA, 20E, and JH III came out to be 0.47, 4.64, 6.47 minute, respectively.

An AB SCIEX Triple Quad™ 6500 (Applied Biosystems) was utilized to carry out triple tandem mass spectrometry. We performed mass spectrometry detection in the positive electrospray ionization mode. The precursor ion was selected as the [M+H] of the analyte. In the multiple-reaction-monitoring (MRM) approach, we set the quantification parameter using mass transitions (precursor ions/product ions), and the ESI ion source temperature was set at 200°C. The remaining mass spectrometric parameters were: DP was 80 V, CE was 30 V, curtain gas had a setting of 35, collisionally activated dissociation was 8, ion-spray voltage was 5500 V, and ion gas 1 and 2 were 20 and 0. Data collection and processing were done using AB SCIEX Analyst 1. 6 (Applied Biosystems).

Hormone analog/mimetics treatment

After being separated and kept in group during 3 to 5 days, adult male and female flies were cold-anesthetized and treated with hormone analog topically on the ventral side of the abdomen (13-14). Methoprene or precocene II was diluted to 1 μg/μL, respectively. Acetone was applied as a vehicle (Control group). As for 20E analog, it was diluted to 1 μg/μL in the ethanol. The ethanol was applied as the control. The

injection volume was 0.5 μL per fly. The entire procedure took within 20 minutes, after a very short recovery time which flies were placed individually in different oviposition dishes to perform the egg-laying assays. To investigate the expression of JH signal-related genes, 10 μL methoprene or precocene II solution in the acetone was added to the surface of ND. Adult female flies were transferred to these vials, respectively. After 8 hours oviposition, ovaries and fat bodies were dissected and collected for RNA extraction.

Statistics

Data were expressed as the means with standard error of the mean (SEM). The figures were administered in the software of GraphPad Prism 8.0.2, R (version 4.2.3), and www.xiantao.love. We assessed the comparison of two groups by One-way ANOVA and two-tailed unpaired *t*-test. Statistical significance was stated as: ns, not significant, $p > 0.05$; *$p < 0.05$, **$p < 0.01$, ***$p < 0.001$.

References

1. Piper, M.D.W., Blanc, E., Leitão-Gonçalves, R., Yang, M., He, X., Linford, N.J., Hoddinott, M.P., Hopfen, C., Soultoukis, G.A., and Niemeyer, C., et al. (2014). A holidic medium for Drosophila melanogaster. NAT METHODS *11*, 100-105.

2. Mu, H., Chen, J., Huang, W., Huang, G., Deng, M., Hong, S., Ai, P., Gao, C., and Zhou, H. (2024). OmicShare tools: A zero-code interactive online platform for biological data analysis and visualization. Imeta *3*, e228.

3. Luo, W., Liu, S., Zhang, W., Yang, L., Huang, J., Zhou, S., Feng, Q., Palli, S.R., Wang, J., and Roth, S., et al. (2021). Juvenile hormone signaling promotes ovulation and maintains egg shape by inducing expression of extracellular matrix genes. P NATL

ACAD SCI USA *118*.

4. Meiselman, M., Lee, S.S., Tran, R.T., Dai, H., Ding, Y., Rivera-Perez, C., Wijesekera, T.P., Dauwalder, B., Noriega, F.G., and Adams, M.E. (2017). Endocrine network essential for reproductive success in Drosophila melanogaster. P NATL ACAD SCI USA *114*, E3849-E3858.

5. Zhang, C., Daubnerova, I., Jang, Y.H., Kondo, S., Zitnan, D., and Kim, Y.J. (2021). The neuropeptide allatostatin C from clock-associated DN1p neurons generates the circadian rhythm for oogenesis. P NATL ACAD SCI USA *118*.

6. Sun, J., Liu, C., Bai, X., Li, X., Li, J., Zhang, Z., Zhang, Y., Guo, J., and Li, Y. (2017). Drosophila FIT is a protein-specific satiety hormone essential for feeding control. NAT COMMUN *8*, 14161.

7. Grmai, L., Michaca, M., Lackner, E., Nampoothiri, V.P.N., and Vasudevan, D. (2024). Integrated stress response signaling acts as a metabolic sensor in fat tissues to regulate oocyte maturation and ovulation. CELL REP *43*, 113863.

8. Kim, B., Kanai, M.I., Oh, Y., Kyung, M., Kim, E., Jang, I., Lee, J., Kim, S., Suh, G.S.B., and Lee, W. (2021). Response of the microbiome–gut–brain axis in Drosophila to amino acid deficit. NATURE *593*, 570-574.

9. Argue, K.J., Yun, A.J., and Neckameyer, W.S. (2013). Early manipulation of juvenile hormone has sexually dimorphic effects on mature adult behavior in Drosophila melanogaster. HORM BEHAV *64*, 589-597.

10. Zhao, B., Hou, Y., Wang, J., Kokoza, V.A., Saha, T.T., Wang, X.L., Lin, L., Zou, Z., and Raikhel, A.S. (2016). Determination of juvenile hormone titers by means of LC-MS/MS/MS and a juvenile hormone-responsive Gal4/UAS system in Aedes aegypti mosquitoes. INSECT BIOCHEM MOLEC *77*, 69-77.

11. Chen, Z., Linse, K.D., Taub-Montemayor, T.E., and Rankin, M.A. (2007).

Comparison of radioimmunoassay and liquid chromatography tandem mass spectrometry for determination of juvenile hormone titers. INSECT BIOCHEM MOLEC *37*, 799-807.

12. Lin, H.H., Cao, D.S., Sethi, S., Zeng, Z., Chin, J., Chakraborty, T.S., Shepherd, A.K., Nguyen, C.A., Yew, J.Y., and Su, C.Y., et al. (2016). Hormonal Modulation of Pheromone Detection Enhances Male Courtship Success. NEURON *90*, 1272-1285.

13. Shi, Y., Liu, T.Y., Jiang, H.B., Liu, X.Q., Dou, W., Park, Y., Smagghe, G., and Wang, J.J. (2019). The Ecdysis Triggering Hormone System, via ETH/ETHR-B, Is Essential for Successful Reproduction of a Major Pest Insect, Bactrocera dorsalis (Hendel). FRONT PHYSIOL *10*, 151.

14. Sugime, Y., Watanabe, D., Yasuno, Y., Shinada, T., Miura, T., and Tanaka, N.K. (2017). Upregulation of Juvenile Hormone Titers in Female Drosophila melanogaster Through Mating Experiences and Host Food Occupied by Eggs and Larvae. ZOOL SCI *34*, 52-57.

Supplementary Figures

Figures S1 to S6

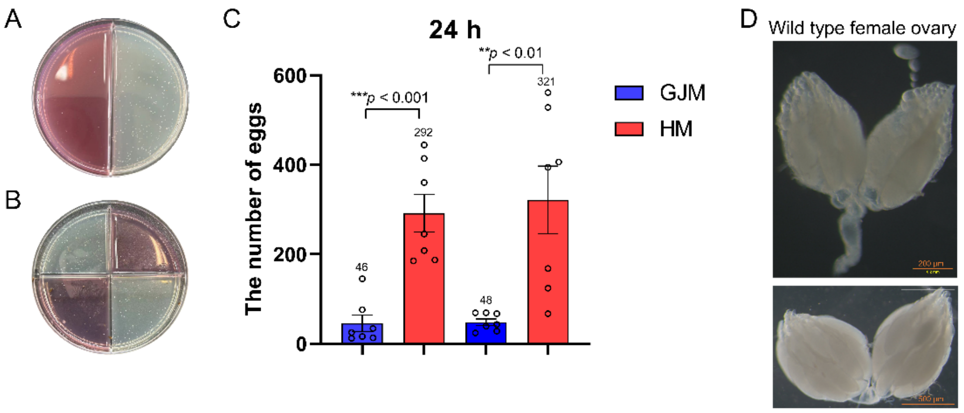

Fig. S1. Eggs laid on different substrates and the morphology of wild type female ovary. A-C, a two-choice egg-laying behavioral assay was performed to comparison between GJM and HM. A-B, the representative observation in the two-choice egg-laying plates. C, the egg production of different substrates within 24 hours. Each container has 100 starved wild-type female flies and 100 starved wild-type male flies. Data from more than 3 experiments (n > 3). D, the ovaries of 3- to 5-day-old wild-type female flies feeding on normal diet (ND). Scale, 200 µm (top), 500 µm (bottom). One-way ANOVA and two-tailed unpaired *t* test were used for the comparison. Data are represented as mean ± SEM. Statistical differences are represented as: ns, not significant, $p > 0.05$; $*p < 0.05$, $**p < 0.01$, $***p < 0.001$.

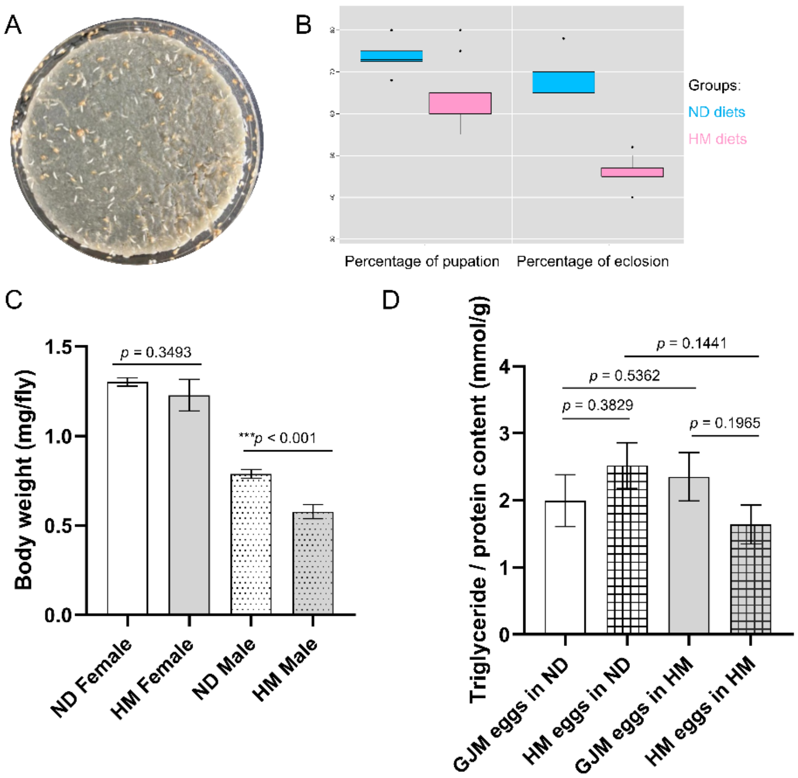

Fig. S2. The development of embryos is independent on egg-laying substrates. A, the growth of eggs laid on HM within 7 days. B, the percentage of pupation and eclosion of eggs fed on ND and HM diets, respectively. C, body weight of adults collected from ND or HM diets. D, lipid content of flies grown up from ND or HM diets. Data from 3 experiments (n = 3). Two-tailed unpaired *t* test were used for the comparison. Data are represented as mean ± SEM. Statistical differences are represented as: ns, not significant, $p > 0.05$; $*p < 0.05$, $**p < 0.01$, $***p < 0.001$.

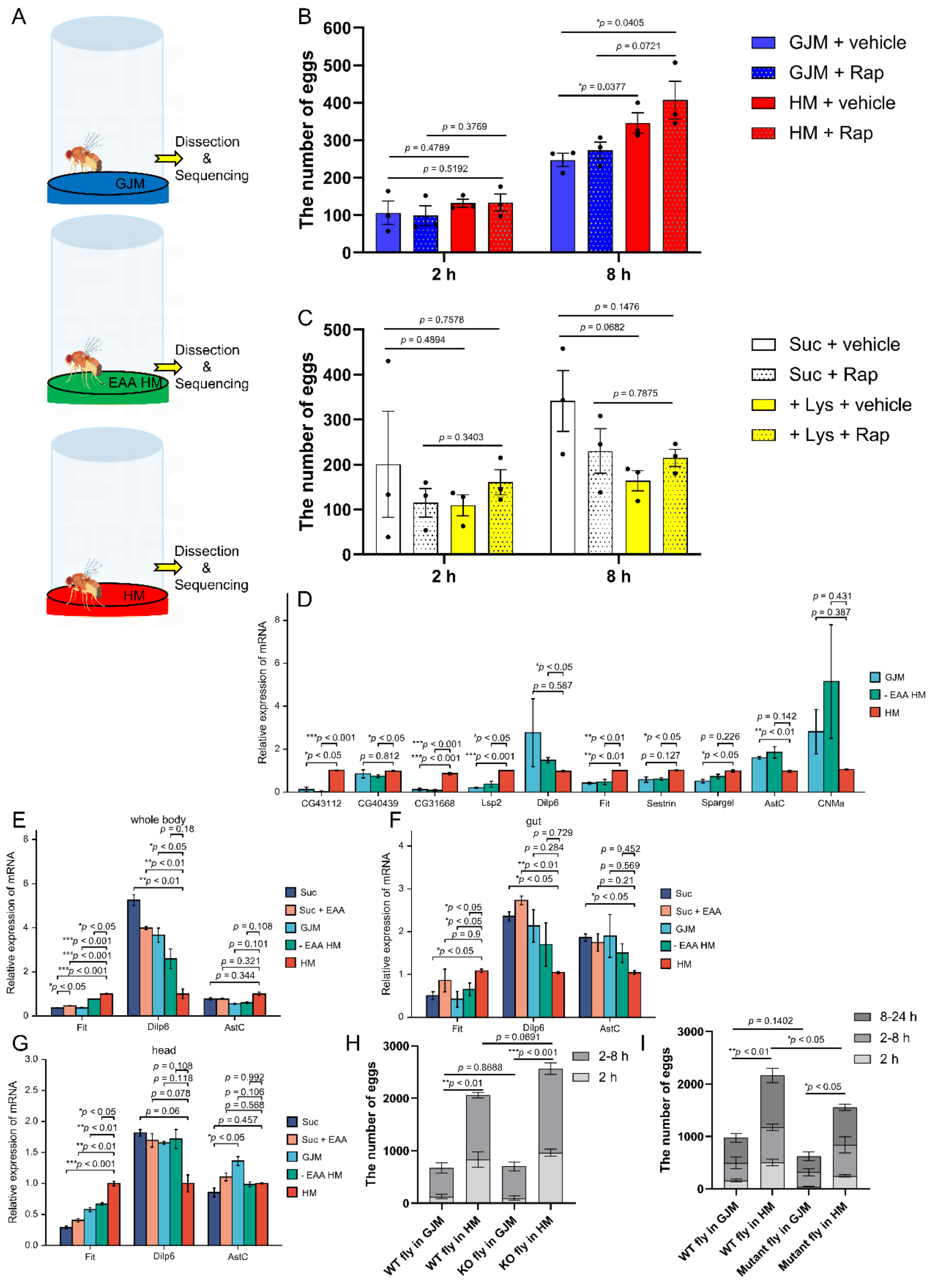

Fig. S3. Different amino acid concentrations induced differential gene expression. Related to Figure 3. A, Illustration of sequencing and qPCR analysis. B-C, the egg production of different substrates within 2 hours and 8 hours, respectively. Each

container has 100 starved wild-type female flies and 100 starved wild-type male flies. Each point represents a separate experiment (n = 3). D, qPCR was determined to validate the transcriptome sequencing data. E-G, three genes (Fit, Dilp6, AstC) differentially expressed derived from whole body, gut, and head. H-I, the eggs produced by wild-type (WT), Fit[81] (KO fly for short), and Dilp6[41] (Mutant fly for short) in the 24-hour period. Data from 3 experiments (n = 3). Two-tailed unpaired t test were used for the comparison. Data are represented as mean ± SEM. Statistical differences are represented as: ns, not significant, $p > 0.05$; $*p < 0.05$, $**p < 0.01$, $***p < 0.001$.

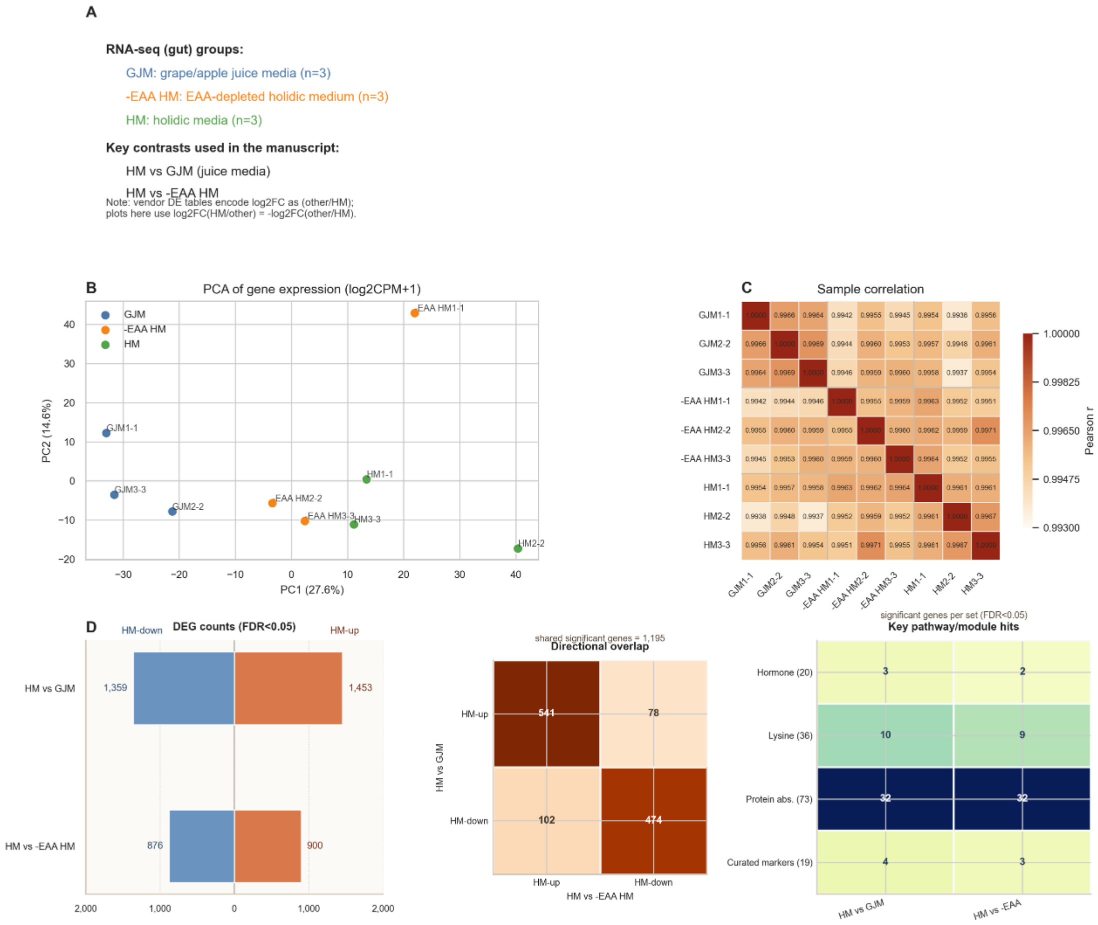

Fig. S4. Gut RNA-seq design, sample quality control, and differential-expression overview. (A) Experimental design showing the three dietary groups used for gut RNA-seq (GJM, -EAA HM, and HM; n = 3 per group) and the two contrasts analyzed in this study (HM vs GJM and HM vs -EAA HM). (B) Principal component analysis of gut transcriptomes based on log2CPM values. (C) Sample-to-sample Pearson correlation heatmap of gut transcriptomes. (D) Summary of differential-expression results across the two contrasts. Left, numbers of significantly upregulated and downregulated genes (FDR < 0.05) in each contrast. Middle, directional overlap of significant genes between contrasts. Right, numbers of significant genes assigned to selected pathways or curated modules, including ko00981 (insect hormone biosynthesis), ko00310 (lysine-related gene set), ko04974 (protein digestion and absorption), and curated JH-related markers. Abbreviations: GJM, grape/apple juice media; HM, holidic medium; -EAA HM, essential amino acid-depleted holidic medium; JH, juvenile hormone.

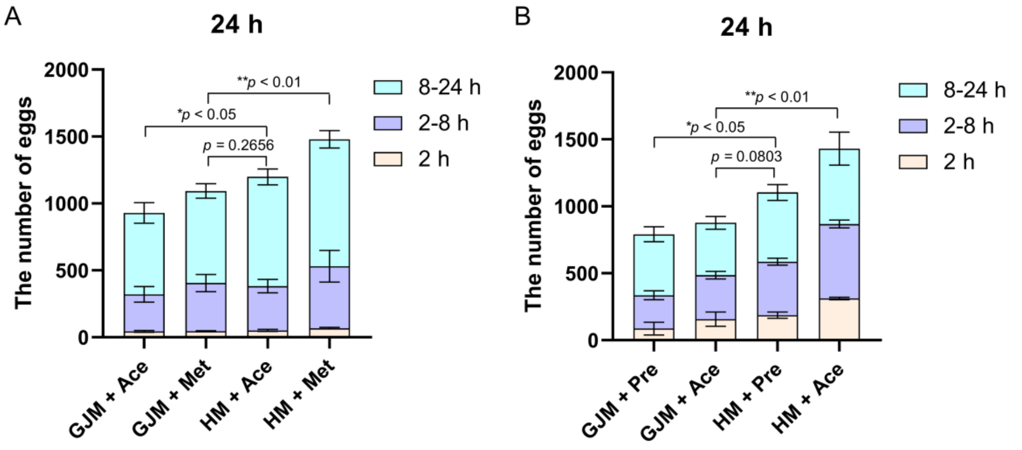

Fig. S5. The relationship between three hormones (20E, ETH and JH) with nutritional modulation of laying phenotype. Related to Figure 5. A-B, eggs laid on GJM and HM by flies treated with Met or Pre within 24 hours, respectively. Acetone was applied as a vehicle (Control group). Met or Pre were applied topically in acetone to the ventral side, respectively. One-way ANOVA and two-tailed unpaired *t* test were used for the comparison. Data are represented as mean ± SEM. Statistical differences are represented as: ns, not significant, $p > 0.05$; $*p < 0.05$, $**p < 0.01$, $***p < 0.001$.

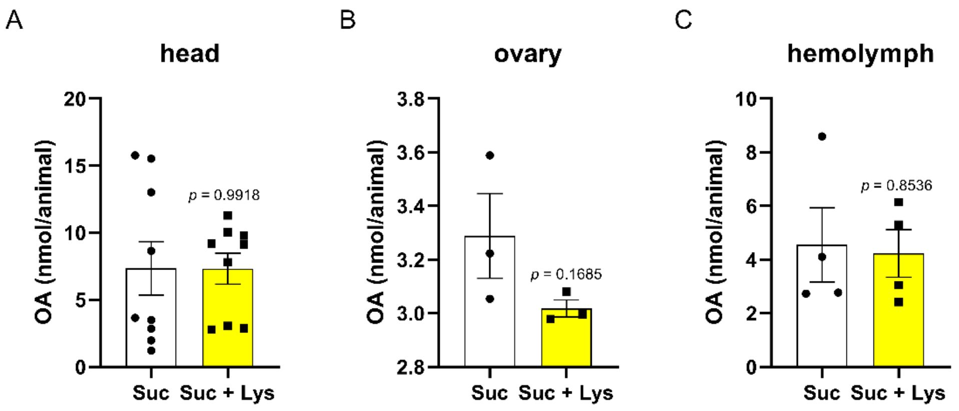

Fig. S6. The content of OA in female head, ovary, and hemolymph using LC-MS/MS. Each container has 50 starved wild-type female flies and 50 starved wild-type male flies. Eggs laid on Suc and Suc + Lys by female flies within 8 hours, respectively. Then females were collected for the dissection. Data from 3 experiments (n = 3). Two-tailed unpaired *t* test were used for the comparison. Data are represented as mean ± SEM. Statistical differences are represented as: ns, not significant, $p > 0.05$; $^*p < 0.05$, $^{**}p < 0.01$, $^{***}p < 0.001$.

Supplementary Tables

Supplementary Table 1 Primer sequence.

Names	Sequences (From 5' to 3')
Rp49-F	AGATCGTGAAGAAGCGCACCAAG
Rp49-R	CACCAGGAACTTCTTGAATCCGG
CG43112-F	AATTGAACGGAAATCGAGATG
CG43112-R	GTGATTGCTTTGACCAGAGGC
CG40439-F	CGAGCATTGGGAGTTCTTGGA
CG40439-R	GCATGTGACGGTATTGGAAAA
CG31668-F	ATTTACCGCAGTTTCTGGGATG
CG31668-R	TACAAGGAAGGCGATGGAGGC
Lsp2-F	TCCGGCTATGCCAGTAACCT
Lsp2-R	GATTCTCCCGGTGCCATTG
Dilp6-F	TCGGTTACGTTCTGCAAGTC
Dilp6-R	CACGGAATACGAACACAGACG
Fit-F	TTGGTGCAGGCCAGGAATAT
Fit-R	CACAGGGCCAGTTGACAGAGT
Sestrin-F	CTCGACTCGATCCCTCCG
Sestrin-R	CAGGTCATCGAGCTCGTCC
Spargel-F	GGATTCACGAATGCTAAATGTGTTCC
Spargel-R	GATGGGTAGGATGCCGCTCAG
AstC-F	GCGCCTATTTGAGGAGTCC
AstC-R	CTTGACGTTTGCTCTCGGG
CNMa-F	GGATTCACGAATGCTAAATGTGTTCC
CNMa-R	CAACAGCAGGAACAGAAGCA

Actin-F	TTCAACACACCCGCCATGTA
Actin-R	AGCCTCCATTCCCAAGAACG
ETH1-F	AGCTGCTTGACAACAACGCTA
ETH1-R	CGAATACTCCACATCTCACAGG
ETH2-F	TTCGCTCTTGGTGGGTCTTG
ETH2-R	CAAAGTTCTCGCCTCGCTTG
Yp1-F	TCCAAGCTGAACACCTATGAGCGT
Yp1-R	TGGTATCGAAGGGCATGTCCAACT
Yp2-F	AGGAAGCAGAACGGTGAACAGGAT
Yp2-R	TACTGCTCAAAGTCCTCGATGGCA
Yp3-F	TACCACGTTGGCCAAATCAAGCAC
Yp3-R	TGGCCGTCTCCACATAGTTGTTCA
Kr-h1-F	GCCCAAATATGAATCCGCTCTACC
Kr-h1-R	GTCGTCGCCCTTGTTCATGTA
LanA-F	CCAGCATCCAGTTCAGCTTC
LanA-R	GACCTTGTGCCATTGGTTGT
LanB1-F	TGGTGCAACAACTGTCCAAG
LanB1-R	TCGGTGTTCGATGCAAGTTC
LanB2-F	GCCAAGAGTGCCATCGATTT
LanB2-R	TGAGCAGGGTCAATGCTGTA
Vkg-F	AGTCCGGCTCCTGTATGATG
Vkg-R	TCGTTGTTCTGGGCAAAGTG
Col4a1-F	TGCTGGTATTCCCGGAGTTT
Col4a1-R	GTTGCCAGTCAAGCCAGTAG
ATF4-F	AGGCCATAGTACCCGCAAAC

ATF4-R	CCGCCTGTTTGTAAGCATCG

Supplementary Table 2 Statistical analysis summary. Related to Figures 1-6 and S1-S6.

Figure	Experiment	*P* value
1B	GJM vs. HM 2 h<br>GJM vs. HM 8 h	$p < 0.0001$<br>$p < 0.0001$
1C	GJM vs. HM 2 h<br>GJM vs. HM 8 h	0.0108; 0.0201<br>$p < 0.001$; $p < 0.001$
1D	Suc vs. HM 2 h<br>Suc vs. HM 8 h	0.0472<br>$p < 0.001$
1E	Suc vs. HM 2 h<br>Suc vs. HM 8 h	0.0493; 0.0438<br>0.0187; 0.0343
1F	– EAA HM vs. HM 2 h<br>– EAA HM vs. HM 8 h	0.0023<br>0.3898
1G	Suc vs. Suc + EAA 2 h<br>Suc vs. GJM 2 h<br>Suc vs. HM 2 h<br>Suc + EAA vs. HM 2 h<br>GJM vs. HM 2 h<br>Suc vs. Suc + EAA 8 h<br>Suc vs. GJM 8 h<br>Suc vs. HM 8 h<br>Suc + EAA vs. HM 8 h<br>GJM vs. HM 8 h	0.0569<br>0.6625<br>$p < 0.0001$<br>$p < 0.0001$<br>$p < 0.0001$<br>0.0273<br>0.7631<br>$p < 0.0001$<br>0.0050<br>$p < 0.0001$

S1C	GJM vs. HM 24 h	0.0002<br>0.0037
S2B	ND vs. HM percentage of pupation<br>ND vs. HM percentage of eclosion	0.0617<br>$p < 0.0001$
S2C	ND vs. HM Female<br>ND vs. HM Male	0.3493<br>0.0004
S2D	GJM vs. HM eggs in ND<br>GJM vs. HM eggs in HM<br>ND vs. HM eggs from GJM<br>ND vs. HM eggs from HM	0.3829<br>0.1965<br>0.5362<br>0.1441
2A	HM vs. – EAA HM<br>HM vs. – Lys HM<br>HM vs. – Ile HM<br>HM vs. – Leu HM<br>HM vs. – Pre HM<br>HM vs. – Thr HM<br>HM vs. – Arg HM<br>HM vs. – His HM<br>HM vs. – Met HM<br>HM vs. – Val HM<br>HM vs. – Trp HM	0.0340<br>0.0314<br>0.0444<br>0.0301<br>0.0835<br>0.2180<br>0.1781<br>0.2397<br>0.4833<br>0.6609<br>0.6524
2B	Suc vs. Suc + Lys<br>Suc vs. Suc + His<br>Suc vs. Suc + Met<br>Suc vs. Suc + Val	0.0184<br>0.1208<br>0.1236<br>0.4299

	Suc vs. Suc + Trp<br>Suc vs. Suc + Thr<br>Suc vs. Suc + EAA<br>Suc vs. Suc + Arg<br>Suc vs. Suc + Ile<br>Suc vs. Suc + Phe<br>Suc vs. Suc + Leu	0.4449<br>0.5474<br>0.4205<br>0.7476<br>0.7939<br>0.9800<br>0.3067
2C	Suc vs. Suc + Lys<br>Suc vs. Suc + Met<br>Suc vs. Suc + EAA<br>Suc vs. Suc + Trp<br>Suc vs. Suc + His<br>Suc vs. Suc + Thr<br>Suc vs. Suc + Phe<br>Suc vs. Suc + Leu<br>Suc vs. Suc + Ile<br>Suc vs. Suc + Val<br>Suc vs. Suc + Arg	0.0342<br>0.9341<br>0.9288<br>0.6613<br>0.5949<br>0.6546<br>0.6072<br>0.5344<br>0.4005<br>0.3101<br>0.3572
2D	Suc vs. Suc + Lys 2 h<br>Suc vs. Suc + Lys 8 h	0.0179<br>0.0226
2E	- Lys HM vs. HM 2 h<br>- AA HM + Lys vs. HM 2 h<br>- Lys HM vs. HM 8 h<br>- AA HM + Lys vs. HM 8 h	0.9938<br>0.1259<br>0.3687<br>0.7386
2F	Suc vs. Suc + Lys 2 h	0.0053

	Suc vs. HM 2 h	$p < 0.0001$
	Suc + Lys vs. HM 2 h	0.0294
	Suc vs. Suc + Lys 8 h	0.0357
	Suc vs. HM 8 h	0.0030
	Suc + Lys vs. HM 8 h	0.0784
3F	GJM vs. HM	0.0050
3G	JHAMT	0.0078
	FPPS	0.0119
	HMGS	0.3162
	HMGCR	0.6953
	Kr-h1	0.3064
S3B	GJM + vehicle vs. GJM + Rap 2 h	0.8610
	HM + vehicle vs. HM + Rap 2 h	0.9506
	GJM + vehicle vs. HM + vehicle 2 h	0.4789
	GJM + Rap vs. HM + Rap 2 h	0.3769
	GJM + vehicle vs. HM + Rap 2 h	0.5192
	GJM + vehicle vs. GJM + Rap 8 h	0.4181
	HM + vehicle vs. HM + Rap 8 h	0.3493
	GJM + vehicle vs. HM + vehicle 8 h	0.0377
	GJM + Rap vs. HM + Rap 8 h	0.0721
	GJM + vehicle vs. HM + Rap 8 h	0.0405
S3C	Suc + vehicle vs. Suc + Rap 2 h	0.5213
	+ Lys + vehicle vs. + Lys + Rap 2 h	0.2314
	Suc + vehicle vs. + Lys + vehicle 2 h	0.4894
	Suc + Rap vs. + Lys + Rap 2 h	0.3403

	Suc + vehicle vs. + Lys + Rap 2 h	0.7578
	Suc + vehicle vs. Suc + Rap 8 h	0.2571
	+ Lys + vehicle vs. + Lys + Rap 8 h	0.1602
	Suc + vehicle vs. + Lys + vehicle 8 h	0.0682
	Suc + Rap vs. + Lys + Rap 8 h	0.7875
	Suc + vehicle vs. + Lys + Rap 8 h	0.1476
S3D	CG43112 GJM vs. HM	0.0025
	- EAA HM vs. HM	0.0015
	CG40439 GJM vs. HM	0.8120
	- EAA HM vs. HM	0.0377
	CG31668 GJM vs. HM	$p < 0.0001$
	- EAA HM vs. HM	$p < 0.0001$
	Lsp2 GJM vs. HM	0.0008
	- EAA HM vs. HM	0.0028
	Dilp6 GJM vs. HM	0.5870
	- EAA HM vs. HM	0.0163
	Fit GJM vs. HM	0.0035
	- EAA HM vs. HM	0.0055
	Sestrin GJM vs. HM	0.1270
	- EAA HM vs. HM	0.0300
	Spargel GJM vs. HM	0.0200
	- EAA HM vs. HM	0.2260
	AstC GJM vs. HM	0.0011
	- EAA HM vs. HM	0.1420
	CNMa GJM vs. HM	0.3870

	- EAA HM vs. HM	0.4310
S3E	Fit Suc vs. Suc + EAA	0.0100
	Suc vs. HM	$p < 0.0001$
	Suc + EAA vs. HM	$p < 0.0001$
	GJM vs. HM	$p < 0.0001$
	- EAA HM vs. HM	0.0008
	Dilp6 Suc vs. Suc + EAA	0.0700
	Suc vs. HM	$p < 0.0001$
	Suc + EAA vs. HM	0.00019
	GJM vs. HM	$p < 0.0001$
	- EAA HM vs. HM	0.1800
	AstC Suc vs. Suc + EAA	0.9999
	Suc vs. HM	0.3440
	Suc + EAA vs. HM	0.3210
	GJM vs. HM	0.1010
	- EAA HM vs. HM	0.1080
S3F	Fit Suc vs. Suc + EAA	0.2745
	Suc vs. HM	0.0043
	Suc + EAA vs. HM	0.9000
	GJM vs. HM	0.0250
	- EAA HM vs. HM	0.0454
	Dilp6 Suc vs. Suc + EAA	0.0666
	Suc vs. HM	0.0003
	Suc + EAA vs. HM	0.0100
	GJM vs. HM	0.2840

	- EAA HM vs. HM	0.7290
	AstC Suc vs. Suc + EAA	0.6431
	Suc vs. HM	0.0010
	Suc + EAA vs. HM	0.2100
	GJM vs. HM	0.5690
	- EAA HM vs. HM	0.4520
S3G	Fit Suc vs. Suc + EAA	0.1600
	Suc vs. HM	0.0014
	Suc + EAA vs. HM	0.0015
	GJM vs. HM	0.0057
	- EAA HM vs. HM	0.0200
	Dilp6 Suc vs. Suc + EAA	0.8500
	Suc vs. HM	0.0600
	Suc + EAA vs. HM	0.0780
	GJM vs. HM	0.1180
	- EAA HM vs. HM	0.1080
	AstC Suc vs. Suc + EAA	0.0600
	Suc vs. HM	0.4570
	Suc + EAA vs. HM	0.5680
	GJM vs. HM	0.1060
	- EAA HM vs. HM	0.9920
S3H	WT GJM vs. HM 2 h	0.0038
	KO GJM vs. HM 2 h	$p < 0.0001$
	GJM WT vs. KO 2 h	0.6531
	HM WT vs. KO 2 h	0.4491

	WT GJM vs. HM 8 h	0.0010
	KO GJM vs. HM 8 h	$p < 0.0001$
	GJM WT vs. KO 8 h	0.8688
	HM WT vs. KO 8 h	0.0691
S3I	WT GJM vs. HM 2 h	0.0028
	Mutant GJM vs. HM 2 h	$p < 0.0001$
	GJM WT vs. Mutant 2 h	0.0028
	HM WT vs. Mutant 2 h	0.0020
	WT GJM vs. HM 8 h	0.0045
	Mutant GJM vs. HM 8 h	0.0204
	GJM WT vs. Mutant 8 h	0.2161
	HM WT vs. Mutant 8 h	0.1538
	WT GJM vs. HM 24 h	0.0037
	Mutant GJM vs. HM 24 h	0.0005
	GJM WT vs. Mutant 24 h	0.1402
	HM WT vs. Mutant 24 h	0.0216
5A	GJM + Met vs. HM + Ace 2 h	0.4911
	GJM + Ace vs. HM + Ace 2 h	0.4903
	GJM + Met vs. HM + Met 2 h	0.0210
	GJM + Met vs. GJM + Ace 2 h	0.9056
	HM + Met vs. HM + Ace 2 h	0.1365
	GJM + Met vs. HM + Ace 8 h	0.7976
	GJM + Ace vs. HM + Ace 8 h	0.4765
	GJM + Met vs. HM + Met 8 h	0.3964
	GJM + Met vs. GJM + Ace 8 h	0.4044

	HM + Met vs. HM + Ace 8 h	0.2957
5B	GJM + Ace vs. HM + Pre 2 h<br>GJM + Pre vs. HM + Pre 2 h<br>GJM + Ace vs. HM + Ace 2 h<br>GJM + Pre vs. GJM + Ace 2 h<br>HM + Pre vs. HM + Ace 2 h<br>GJM + Ace vs. HM + Pre 8 h<br>GJM + Pre vs. HM + Pre 8 h<br>GJM + Ace vs. HM + Ace 8 h<br>GJM + Pre vs. GJM + Ace 8 h<br>HM + Pre vs. HM + Ace 8 h	0.6670<br>0.1477<br>0.0593<br>0.3556<br>0.0075<br>0.3375<br>0.0110<br>0.0144<br>0.0837<br>0.0266
5C	Suc vs. Suc + Lys	0.0173
5D	Suc vs. Suc + Lys	0.0934
5E	Suc vs. Suc + Lys	0.0333
5F	Suc vs. Suc + Lys	0.0202
5G	ETH1<br>ETH2	0.0230<br>0.0369
5H	Suc + 20E vs. Lys + EtOH<br>Suc + Met vs. Lys + EtOH<br>Suc + EtOH vs. Lys + EtOH<br>Suc + 20E vs. Lys + 20E<br>Suc + Met vs. Lys + Met<br>Suc + 20E vs. Suc + EtOH<br>Suc + Met vs. Suc + EtOH<br>Lys + 20E vs. Lys + EtOH	0.1058<br>0.1498<br>0.6094<br>0.2306<br>0.4371<br>0.0346<br>0.0463<br>0.0091

	Lys + Met vs. Lys + EtOH	0.0608
5I	Suc + 20E vs. Lys + EtOH<br><br>Suc + Met vs. Lys + EtOH<br><br>Suc + EtOH vs. Lys + EtOH<br><br>Suc + 20E vs. Lys + 20E<br><br>Suc + Met vs. Lys + Met<br><br>Suc + 20E vs. Suc + EtOH<br><br>Suc + Met vs. Suc + EtOH<br><br>Lys + 20E vs. Lys + EtOH<br><br>Lys + Met vs. Lys + EtOH	0.6845<br><br>0.7510<br><br>0.0420<br><br>0.2232<br><br>0.4212<br><br>0.0159<br><br>0.0677<br><br>0.1382<br><br>0.5293
S5A	GJM + Met vs. HM + Ace 24 h<br><br>GJM + Ace vs. HM + Ace 24 h<br><br>GJM + Met vs. HM + Met 24 h<br><br>GJM + Met vs. GJM + Ace 24 h<br><br>HM + Met vs. HM + Ace 24 h	0.2656<br><br>0.0226<br><br>0.0048<br><br>0.0049<br><br>0.0566
S5B	GJM + Ace vs. HM + Pre 24 h<br><br>GJM + Pre vs. HM + Pre 24 h<br><br>GJM + Ace vs. HM + Ace 24 h<br><br>GJM + Pre vs. GJM + Ace 24 h<br><br>HM + Pre vs. HM + Ace 24 h	0.0803<br><br>0.0505<br><br>0.0073<br><br>0.5672<br><br>0.0502
6A	Yp1 + Met vs. + Ace<br><br>Yp1 + Ace vs. + Pre<br><br>Yp2 + Met vs. + Ace<br><br>Yp2 + Ace vs. + Pre<br><br>Yp3 + Met vs. + Ace	0.4622<br><br>0.0027<br><br>0.0040<br><br>$p < 0.001$<br><br>0.1061

	Yp3 + Ace vs. + Pre	0.0054
	Kr-h1 + Met vs. + Ace	0.1480
	Kr-h1 + Ace vs. + Pre	0.0026
	LanA + Met vs. + Ace	0.0978
	LanA + Ace vs. + Pre	$p < 0.001$
	LanB1 + Met vs. + Ace	0.0144
	LanB1 + Ace vs. + Pre	0.0118
	LanB2 + Met vs. + Ace	0.0018
	LanB2 + Ace vs. + Pre	0.0165
6B	Kr-h1 + Met vs. + Ace	$p < 0.001$
	Kr-h1 + Ace vs. + Pre	0.0116
	Vkg + Met vs. + Ace	0.0012
	Vkg + Ace vs. + Pre	0.0212
	Col4a1 + Met vs. + Ace	0.0456
	Col4a1 + Ace vs. + Pre	0.0054
6C	Yp1	0.5902
	Yp2	0.0350
	Yp3	0.0348
	Kr-h1	0.0475
	LanA	0.0417
	LanB1	0.0247
	LanB2	0.6191
	Vkg	0.0293
	Col4a1	0.3587
6D	ATF4	0.0399

S6A	head	0.9918
S6B	ovary	0.1685
S6C	hemolymph	0.8536
